# Identification of desalination and wind power plants sites using *m*-polar fuzzy Aczel–Alsina aggregation information

**DOI:** 10.1038/s41598-023-50397-6

**Published:** 2024-01-03

**Authors:** Zia Ur Rahman, Ghous Ali, Muhammad Asif, Yufeng Chen, Muhammad Zain Ul Abidin

**Affiliations:** 1 School of Mathematical Sciences, Zhejiang Normal University, Jinhua, China; 2 School of Management and Economics, Zhejiang Normal University, Jinhua, China; 3 Division of Science and Technology, Department of Mathematics, University of Education, Lahore, Pakistan; 4 Faculty of Sciences, The Superior University, Lahore, Pakistan

**Keywords:** Applied mathematics, Computational science, Hydrology, Ocean sciences

## Abstract

Real-world decision-making problems often include multi-polar uncertainties dependent on multi-dimensional attributes. The *m*-polar fuzzy (*m*F) sets can efficiently handle such multi-faceted complications with T-norm based weighted aggregation techniques. The Aczel–Alsina T-norms offer comparatively flexible and accurate aggregation than the other well-known T-norm families. Consequently, this work introduced novel *m*F Aczel–Alsina aggregation operators (AOs), including weighted averaging (*m*FAAWA, *m*FAAOWA, *m*FAAHWA) and weighted geometric (*m*FAAWG, *m*FAAOWG, *m*FAAHWG) AOs. The fundamental properties, including boundedness, idempotency, monotonicity, and commutativity are investigated. Based on the proposed AOs, a decision-making algorithm is developed and implemented to solve two detailed multi-polar site selection problems (for desalination plant and for wind-power plant). Finally, a comparison with *m*F Dombi and *m*F Yager AOs reveals that different T-norm based AOs may yeild different solutions for the same problem.

## Introduction

Multi-criteria decision-making (MCDM) is the process of making decisions about various alternatives affected by multiple decision parameters. Human beings have ever been making decisions in complicated scenarios by considering the trade-offs of conflicting attributes affecting the situation. However, the first appropriate MCDM method can be dated back to 1972 as the Benjamin Franklin’s papers^1^ considering his “moral or prudential algebra” in his letter to Priestley. His method considered balancing the pros and cons of two conflicting attributes on two sides of a piece of paper, to give them preferences and making difficult decisions accordingly. Later, more detailed, methodological, and complicated MCDM approaches were established and utilized in various disciplines. K$\ddot{o}$ksalan et al.^2^ discussed more than 50 years long MCDM developments till the 21st century listing many foundational contributors to this field. MCDM accounts for the solutions of a huge set of ever expanding problems, from diverse domains, with the help of many different approaches. For instance, Ozernoy^3^ proposed a framework for selecting the most appropriate MCDM approach in decision support systems. Wang and Triantaphyllou^4^ analyzed various real-life MCDM problems and identified irregularities in the rankings of alternatives when applying different MCDM methods.

Different MCDM techniques are suitable for different circumstances and often new techniques generalize the previous ones. The literature on MCDM approaches reveals many studies based on both crisp and fuzzy logics. A crisp set backed by a binary-valued classification is adequate for dealing with certain information defined with sharp boundaries. However, this binary classification fails in uncertain scenarios. The fuzzy sets (FSs) based on fuzzy logic proved helpful in such cases by declaring partial memberships from [0, 1] to objects depicting uncertain behavior. This FS concept was introduced independently by Zadeh^5^ as an extension of the crisp set theory (generalization of the binary-valued logic). In 1970, Bellman and Zadeh^6^ discussed decision-making in a fuzzy environment by taking into account the fuzzy constraints appearing in alternatives or criterion during the decision-making process. From the inception of FS theory to the present day, it has played a vital role in numerous areas, including medicine, engineering, management science, artificial intelligence, operations research, computing, robotics, pattern recognition, and so on. Many extensions of the fuzzy theory have been observed, for example, intuitionistic FSs (IFSs)^7^ and Pythagorean FSs (PFSs)^8^ (both containing two separate fuzzy degrees for membership degree (MD) and non-membership degree (NMD) with their particular summation restrictions). In 1994, Zhang^9^ introduced another extension of FSs, namely, bipolar FS (BFS) theory. The main idea behind this contribution is the bipolar information existing about a particular set. This bipolar information includes the positive and negative sides of an aspect (property and its counter-property) like good and bad, young and dull, fair and unfair, etc. To depict the notion of bipolarity, BFSs consider memberships from closed interval [$-1$, 1], such that 0 indicates irrelevance to the criteria, MD from [$-1$, 0) indicates satisfaction of object with the corresponding counter-criteria, and MD from (0, 1] depicts satisfaction with the considered criteria. Till date, several significant researches have been conducted on this theory to make more enhanced decision-making methods (see Refs.^10–13^). Apart from the research on decision models, fuzzy theory has spread its roots in many technical fields and has been applied to many future-oriented applications. Recently, Talpur et al.^14^ discussed the deep neuro-fuzzy systems, their applications, challenges, and possibilities. Deveci et al.^15^ utilized an interval type-2 fuzzy set based method for saving the environment by improvising the sustainable vehicle shredding facilities.

Various daily-life scenarios are governed by multi-polar attributes affecting multi-faceted characteristics of the alternatives which leads to the emergence of multi-polar information unsolvable by conventional mathematical tools such as crisp set theory, FS theory, intuitionistic FS theory, and bipolar FS theory. Contemporary researchers are increasingly recognizing the substantial role of multi-polarity in a vast range of domains, spanning from medical sciences to engineering, management to neural fuzzy developments. For instance, consider the realm of information technology, where multi-polar technology can be employed to analyze complex information systems with varying attributes like latency, bandwidth, radio frequency, and network range. In neurobiology, the interconnectedness of neurons in the brain gather data from different other neurons, considering a multi-polar information gathering procedure. Likewise, within a social network, individuals may exhibit varying levels of effectiveness in their trading relationships, proactiveness, and sociability, all of which involve multi-polar data. To address these multi-polar scenarios, Chen et al.^16^ initiated the theory of *m*-polar fuzzy (*m*F) sets, specifically formulated to tackle multi-polarity in datasets across diverse domains of modern sciences. These *m*F sets ($m\ge 2$ generalizing bipolar fuzzy sets) consider separate fuzzy memberships for *m* distinct dimensions of a particular criteria/characteristic/attribute. Some recent applications include *m*F algorithm applied to selection of non-tradional machining process^17^, *m*F networks utilized for product manufacturing problems^18^, and more.

These days, MCDM methods based on aggregation operators (AOs) are playing an increasingly significant role in various fields, including engineering, medicine, economics, environmental sciences, and more. Consequently, several decision-making techniques based on AOs have been introduced for MCDM to enhance the precision of optimal decisions, and they continue to evolve for further advancements. For example, Xu^19^ explored some AOs based on intuitionistic FSs, including weighted, ordered weighted, and hybrid weighted averaging AOs (for more details on intuitionistic FS-based AOs, see Refs.^20,21^). Garg et al.^22^ presented Schweizer-Sklar prioritized AOs for IFSs with their decision-making applications. Peng and Yang^23^ presented certain basic notions of Pythagorean FS-based AOs within an interval-valued context. Over the last decade, there have been several noteworthy studies focused on the aggregation of bipolar data using leveraging established operations. For instance, Wei et al.^24^ introduced Hamacher aggregation operators tailored to bipolar data and investigated their applications in MCDM. Jana et al.^25^ devised AOs based on bipolar information utilizing Dombi’s operations, effectively addressing practical problems in daily-life. Moreover, Jana et al.^26^ pioneered the advancement of bipolar fuzzy Dombi prioritized AOs with their innovative contributions. Subsequently, experts have investigated the aggregation of *m*F datasets using different established AOs. For instance, Waseem et al.^27^ proposed *m*F Hamacher AOs and implemented them to solve MCDM problems. Khameneh and Kilicman^28^ developed *m*F soft weighted AOs, which were effectively used to address MCDM problems. Akram et al.^29^ presented *m*F Dombi AOs and explored their applications in MCDM. Later, Naz et al.^30^ introduced innovative 2-tuple linguistic bipolar fuzzy Heronian mean AOs for group decision-making. Recently, Ali et al.^31^ proposed specific arithmetic and geometric AOs for the aggregation of *m*F datasets with Yager’s operations. For more MCDM applications of AOs, the readers may visit^32–35^.

In early 1980s, Aczel and Alsina^36^ introduced the Aczel–Alsina *t*-norm (TN) and *t*-conorm (TCoN) as modified forms of the algebraic norms. Among the other TNs (and TCoNs), Aczel–Alsina TN (and TCoN) provide more accurate decisions. To demonstrate this, Farahbod and Eftekhari^37^ classified nine different TN-based AOs on the basis of their aggregating accuracy in analyzing 12 different datasets. The Aczel–Alsina operators dominated in the analysis showing minimum error as compared to other operators. Consequently, recent researches have focused on Aczel–Alsina TNs and TCoNs based AOs for all the above discussed theories. Mahmood et al.^38^ presented Aczel–Alsina TN and TCoN based AOs in bipolar complex fuzzy environment and explored their application in selecting the best operating system (see also, Mahmood and Ali^39^). Akram et al.^40^ used generalized orthopair fuzzy Azcel-Alsina AOs for energy resource selection. Ali et al.^41^ introduced intuitionistic fuzzy soft Aczel–Alsina AOs. Wang et al.^42^ utilized Aczel–Alsina based Hamy-Mean AOs for T-spherical fuzzy MCDM. Some other contributions include^43–45^. Considering this efficiency and applicability of Aczel–Alsina TN and TCoN, and the need for multi-polar fuzzy aggregation, this work focuses on the development of *m*F set-based Aczel–Alsina AOs. The motivations for the proposed work are listed below:

1. Aczel–Alsina TN/TCoN are more flexible and accurate as compared to other TNs/TCoNs in their aggregation capabilities. Existing literature^37^ clearly demonstrates this dominating accuracy of the Aczel Alsina AOs.
2. Real-world decision-making problems like site selection for a massive project are often based on multi-faceted information in the form of multi-agent, multi-attribute, multi-polar uncertainties. Crisp, fuzzy, and bipolar fuzzy models fail to solve such multi-faceted decision-making problems effectively.
3. Multi-polar fuzzy sets offer solutions to multi-polar uncertainties by considering multiple distinct aspects of an alternative. This provides much suited, accurate, and flexible decision-making for multi-faceted scenarios, as compared to other decision models.
4. The combination of aggregation capabilities of Aczel–Alsina TN/TCoN and multi-polar uncertainty modeling of m-polar fuzzy sets can provide accurate decision-making for multi-polar uncertain situations. The existing literature lacks work on this powerful combination.

On the basis of these motivations, the proposed work focuses on the development of multi-polar fuzzy (*m*F) Aczel–Alsina AOs and demonstration of their decision-making capabilities. The following list accounts for this work’s key contributions.

1. Development of novel Aczel–Alsina AOs for *m*F information including weighted averaging AOs (*m*FAAWA, *m*FAAOWA, *m*FAAHWA) and weighted geometric AOs (*m*FAAWG, *m*FAAOWG, *m*FAAHWG).
2. Detailed analysis of the fundamental properties of proposed AOs including idempotency, monotonicity, boundedness, and commutativity.
3. Development of a working decision-making algorithm for multi-polar information based on *m*FAAWA and *m*FAAWG AOs.
4. Deep investigation of two model multi-polar site selection problems (for desalination plant and for wind-power plant) with proposed techniques, and ranking of available sites against multi-polar attributes under the novel decision-making algorithm.
5. Discussion on the advantageous and limiting features of proposed techniques in addition to a comparative analysis with *m*F Yager AOs^31^ and *m*F Dombi AOs^29^.

The upcoming work is structured as follows:

1. Section “Preliminaries” revisits some *m*F concepts and recalls the Aczel–Alsina TN and T-CoN.
2. Section “*m*F Aczel–Alsina AOs” proposes novel *m*F Aczel–Alsina averaging and geometric AOs (weighted, ordered weighted, hybrid weighted). This section further analyses fundamental properties of proposed AOs.
3. Section “Applications to MCDM with *m*F information” introduces a unique MCDM algorithm based on *m*F Aczel–Alsina AOs. Under the offered methodologies, detailed modeling and solutions to two multi-polar site selection problems (for a desalination facility and a wind-power plant) are presented.
4. Section “Discussion” compares the proposed AOs with *m*F Yager AOs^31^ and *m*F Dombi AOs^29^. The advantages and limitations of the proposed work are shortly discussed.
5. Section “Conclusions and future plans” gives the conclusive remarks and future directions.

## Preliminaries

The following definition recalls multi-polar fuzzy (*m*F) sets:

### Definition 2.1

^16^A *m*F set on a universal set *U* is a mapping $\eta :U\rightarrow [0,1]^m$. The belongingness of each alternative is given by

$$\begin{aligned} \eta (\mu )=\{P_1\circ \eta (\mu ),P_2\circ \eta (\mu ), \ldots P_m\circ \eta (\mu )\}, \end{aligned}$$

such that $P_i\circ \eta :[0,1]\rightarrow [0,1]$ is the $i^{\textrm{th}}$ projection mapping.

Following define the notions of score and accuracy functions of a *m*F number $\eta$.

### Definition 2.2

^27^The score function of a *m*F number $\eta =\{P_1\circ \eta ,P_2\circ \eta , \ldots P_m\circ \eta \}$ is defined as:

$$S(\eta ) = \frac{1}{m}\left( {\sum\limits_{{i = 1}}^{m} {P_{i} \circ \eta } } \right),\quad S(\eta \in [0,1]).$$

### Definition 2.3

^27^The accuracy function of a *m*F number $\eta =\{P_1\circ \eta ,P_2\circ \eta , \ldots P_m\circ \eta \}$ is defined as:

$$\begin{aligned} H(\eta )=\dfrac{1}{m}{\sum _{i=1}^{m}(-1)^{i+1}(P_i\circ \eta -1),H(\eta )\in [-1,1]}. \end{aligned}$$

here, $$S(\eta )\in [0,1]$$ and $$H(\eta )\in [-1,1]$$.

### Definition 2.4

^27^For $\eta _1=\{P_1\circ \eta _1,P_2\circ \eta _1, \ldots P_m\circ \eta _1\}$ and $\eta _2=\{P_1\circ \eta _2,P_2\circ \eta _, \ldots P_m\circ \eta _2\}$ representing two *m*FNs with score function $S(\cdot )$ and accuracy function $H(\cdot )$, the following are satisfied:

1. $$\eta _1<\eta _2$$, if $$S(\eta _1)<S(\eta _2)$$,
2. $$\eta _1>\eta _2$$ if $$S(\eta _1)>S(\eta _2),$$
3. $$\eta _1=\eta _2$$ if $$S(\eta _1)=S(\eta _2)$$ and $$H(\eta _1)=H(\eta _2)$$,
4. $$\eta _1<\eta _2$$ if $$S(\eta _1)=S(\eta _2)$$ but $$H(\eta _1)<H(\eta _2)$$,
5. $$\eta _1>\eta _2$$ if $$S(\eta _1)=S(\eta _2)$$ but $$H(\eta _1)>H(\eta _2)$$.

Next, some basic operation for *m*FNs are defined and their properties are discussed.

### Definition 2.5

Let $\eta =\{P_1\circ \eta ,P_2\circ \eta , \ldots P_m\circ \eta \}$, $\eta _1=\{P_1\circ \eta _1,P_2\circ \eta _1, \ldots P_m\circ \eta _1\}$, and $\eta _2=\{P_1\circ \eta _2,P_2\circ \eta _, \ldots P_m\circ \eta _2\}$ be the *m*FNs and $\alpha$ be a scalar. Then

1. $$\eta _1\bigoplus \eta _2={(P_1\circ \eta _1+P_1\circ \eta _2-P_1\circ \eta _1.P_2\circ \eta _2,\ldots ,P_m\circ \eta _1+P_m\circ \eta _2-P_m\circ \eta _1.P_m\circ \eta _2)}$$.
2. $$\eta _1\bigotimes \eta _2={(P_1\circ \eta _1.P_1\circ \eta _2,\ldots ,P_m\circ \eta _1.P_m\circ \eta _2)}$$.
3. $$\alpha \eta =((1-(1-P_1\circ \eta )^\alpha ),\ldots ,1-(1-P_m\circ \eta )^\alpha ), \alpha >0$$.
4. $$(\eta )^\alpha =((P_1\circ \eta )^\alpha ,\ldots ,(P_m\circ \eta )^\alpha ), \alpha >0$$.
5. $$(\eta )^c=(1-P_1\circ \eta ,\ldots ,P_m\circ \eta )$$
6. $\eta _1\subseteq \eta _2$, if and only if $P_1\circ \eta _1\le P_1\circ \eta _2,\ldots ,P_m\circ \eta _1\le P_m\circ \eta _2$.
7. $$\eta _1\cup \eta _2=(\max (P_1\circ \eta _1,P_1\circ \eta _2),\ldots ,\max (P_m\circ \eta _1,P_m\circ \eta _2)).$$
8. $$\eta _1\cap \eta _2=(\min (P_1\circ \eta _1,P_1\circ \eta _2),\ldots ,\min (P_m\circ \eta _1,P_m\circ \eta _2)).$$

### Theorem 2.1

Consider two *m*FNs $\eta _1=\{P_1\circ \eta _1,P_2\circ \eta _1, \ldots P_m\circ \eta _1\}$ and $\eta _2=\{P_1\circ \eta _2,P_2\circ \eta _2, \ldots P_m\circ \eta _2\}$ with $\alpha$, $\alpha _1$, $\alpha _2>0$, then

1. $$\eta _1\bigoplus \eta _2=\eta _2\bigoplus \eta _1$$,
2. $$\eta _1\bigotimes \eta _2=\eta _2\bigotimes \eta _1$$,
3. $$\alpha (\eta _1\bigoplus \eta _2)=\alpha (\eta _1)\bigoplus (\eta _2)$$,
4. $$(\eta _1\bigotimes \eta _2)^\alpha =(\eta _1)^\alpha \bigotimes (\eta _2)^\alpha$$,
5. $$\alpha _1\eta _1\bigoplus \alpha _2\eta _1=(\alpha _1+\alpha _2)\eta _1$$,
6. $$(\eta _1)^{\alpha _1}\bigotimes (\eta _1)^{\alpha _2}=(\eta _1)^{\alpha _1+\alpha _2}$$,
7. $$((\eta _1)^{\alpha _1})^{\alpha _2}=(\eta _1)^{\alpha _1\alpha _2}.$$

Coming definition recalls the Aczel–Alsina TN and TCoN introduced by Aczel and Alsina^36^ in 1980.

### Definition 2.6

The Aczel–Alsina TN $\Psi$ and TCoN $\Psi ^*$ are defined as:

$$\begin{aligned} & \Psi (x,y) = x \bigotimes y = \left( {\frac{1}{{e^{{\{ ( - ln(x))^{p} + ( - ln(y))^{p} \} ^{{1/p}} }} }}} \right), \\ & \Psi ^{*} (x,y) = x \bigoplus y = \left( {1 - \frac{1}{{e^{{\{ ( - ln(1 - x))^{p} + ( - ln(1 - y))^{p} \} ^{{1/p}} }} }}} \right), \\ \end{aligned}$$

where $$1<p<\infty$$ and $$x,y\in [0,1]$$.

## *m*F Aczel–Alsina AOs

This section firstly gives Aczel–Alsina operations for *m*F numbers via Aczel–Alsina TN and TCoN, and then proceeds to development of *m*F Aczel–Alsina averaging and geometric AOs while discussing their basic properties.

### *m*F Aczel–Alsina operations

Let there be three *m*F numbers $\eta _1=\{P_1\circ \eta _1,P_2\circ \eta _1, \ldots P_m\circ \eta _1\}, \eta _2=\{P_1\circ \eta _2,P_2\circ \eta _2, \ldots P_m\circ \eta _2\}$ and $\eta =\{P_1\circ \eta ,P_2\circ \eta , \ldots P_m\circ \eta \}$. Then for $1<p<\infty$,

$$\begin{aligned} \eta _1\bigoplus \eta _2&=\Big (1-\dfrac{1}{e^{\{(-ln(1-P_1\eta _1))^p+(-ln(1-P_1\eta _2))^p\}^{1/p}}},\dots ,\\&~~~~~~1-\dfrac{1}{e^{\{(-\ln (1-P_m\eta _1))^p+(-ln(1-P_m\eta _2))^p\}^{1/p}}}\Big ),\\ \eta _1\bigotimes \eta _2&=\left( \dfrac{1}{e^{\{(-lnP_1\eta _1)^p+(-lnP_1\eta _2)^p\}^{1/p}}},\ldots ,\dfrac{1}{e^{\{(-lnP_m\eta _1)^p+(-lnP_m\eta _2)^p\}^{1/p}}}\right) ,\\ \alpha \eta&=\left( 1-\dfrac{1}{e^{\{\alpha (-ln(1-P_1\eta )^p)\}^{1/p}}},\ldots ,1-\dfrac{1}{e^{\{\alpha (-ln(1-P_m\eta )^p)\}^{1/p}}}\right) ,\\ \eta ^\alpha&=\left( \dfrac{1}{e^{\{\alpha (-lnP_1\eta )^p\}^{1/p}}},\ldots ,\dfrac{1}{e^{\{\alpha (-lnP_m\eta )^p\}^{1/p}}}\right) . \end{aligned}$$

### *m*F Aczel–Alsina weighted averaging AOs

This subsection establishes *m*FAAWA, *m*FAAOWA and *m*FAAHWA AOs and investigates some of their basic properties. In the coming developments, $\gamma$=$\left( \gamma _1,\gamma _2,\gamma _3,\ldots ,\gamma _n\right)$ acts as the weight vector with $\gamma _k>0$ and $\sum _{k=1}^{n}\gamma _k=1$.

#### Definition 3.1

Let $\eta _k=\left( P_1\circ \eta _k,P_2\circ \eta _k,\ldots ,P_m\circ \eta _k\right)$ be the collection of ‘*n*’ *m*F numbers where $k=1,2,\ldots ,n$. Then a mapping from $\eta ^n$ to $\eta$ defines the *m*FAAWA AO as:

$$\begin{aligned} mFAAWA_\gamma \left( \eta _1,\eta _2,\ldots ,\eta _n\right) =\sum _{k=1}^{n}\left( \gamma _k\eta _k\right) . \end{aligned}$$

#### Theorem 3.1

Let $\eta _k=\left( P_1\circ \eta _k,P_2\circ \eta _k,\ldots ,P_m\circ \eta _k\right)$ be the collection of ‘*n*’ *m*F numbers where $k=1,2,\ldots ,n$. The aggregation of these *m*F numbers by *m*FAAWA AO is given as:

1 $$\begin{aligned} mFAAWA_\gamma \left( \eta _1,\eta _2,\ldots ,\eta _n\right) =&\sum _{k=1}^{n}\left( \gamma _k\eta _k\right) \nonumber \\ =&\Big (1-\dfrac{1}{e^{\{\sum _{k=1}^{n}\gamma _k(-ln(1-P_1\eta _k))^p\}^{1/p}}},\ldots ,\nonumber \\ {}&1-\dfrac{1}{e^{\{\sum _{k=1}^{n}\gamma _k(-ln(1-P_m\eta _k))^p\}^{1/p}}}\Big ). \end{aligned}$$

#### Proof

By using induction method, for $n=1$ and $\gamma _1=1$,

$$\begin{aligned} mFAAWA_\gamma \left( \eta _1,\eta _2,\ldots ,\eta _n\right)&=\left( \gamma _1\eta _1\right) =\eta _1,\\&=\left( 1-\dfrac{1}{e^{\{(-ln(1-P_1\eta _1))^p\}^{1/p}}},\ldots ,1-\dfrac{1}{e^{\{(-ln(1-P_m\eta _1))^p\}^{1/p}}}\right) . \end{aligned}$$

Hence, the result obtained by *m*FAAWA operator satisfies Equation (1) for $n=1$. Now, suppose that Eq. (1) is true for $n=t$, then

$$\begin{aligned} mFAAWA_\gamma \left( \eta _1,\eta _2,\ldots ,\eta _n\right) =&\sum _{k=1}^{t}\left( \gamma _k\eta _k\right) ,\\ =&\left( 1-\dfrac{1}{e^{\{\sum _{k=1}^{t}\gamma _k(-ln(1-P_1\eta _k))^p\}^{1/p}}},\ldots ,1-\dfrac{1}{e^{\{\sum _{k=1}^{t}\gamma _k(-ln(1-P_m\eta _k))^p\}^{1/p}}}\right) ,\\ \end{aligned}$$

Next it needs to be shown that the theorem holds for $n=t+1$. By putting $n=t+1$ in Eq. (1),

$$\begin{aligned} mFAAWA_\gamma \left( \eta _1,\eta _2,\ldots ,\eta _n\right) =&\sum _{k=1}^{t+1}\left( \gamma _k\eta _k\right) , \\=&\sum _{k=1}^{t}\left( \gamma _k\eta _k\right) \bigoplus \left( \gamma _{t+1}\circ \eta _{t+1}\right) , \\=&\left( 1-\dfrac{1}{e^{\{\sum _{k=1}^{t}\gamma _k(-ln(1-P_1\eta _k))^p\}^{1/p}}},\ldots ,1-\dfrac{1}{e^{\{\sum _{k=1}^{t}\gamma _k(-ln(1-P_m\eta _k))^p\}^{1/p}}}\right) , \\ {}&~~\bigoplus \left( 1-\dfrac{1}{e^{\{\gamma _{t+1}(-ln(1-P_1\eta _{t+1}))^p\}^{1/p}}},\ldots ,1-\dfrac{1}{e^{\{\gamma _{t+1}(-ln(1-P_m\eta _{t+1}))^p\}^{1/p}}}\right) , \\=&\left( 1-\dfrac{1}{e^{\{\sum _{k=1}^{t+1}\gamma _k(-ln(1-P_1\eta _k))^p\}^{1/p}}},\ldots ,1-\dfrac{1}{e^{\{\sum _{k=1}^{t+1}\gamma _k(-ln(1-P_m\eta _k))^p\}^{1/p}}}\right) . \end{aligned}$$

Hence, Eq. (1) verifies for $n=t+1$, which proves the theorem by induction method. $\square$

#### Example 3.1

Let $\eta _1$=$\left( 0.52,0.16,0.37\right)$, $\eta _2$=$\left( 0.31,0.80,0.25\right)$ and $\eta _3=\left( 0.73,0.91,0.16\right)$ be 3F numbers and $\gamma =\left( 0.25,0.45,0.30\right)$ be the corresponding weight vector. Then

2 $$\begin{aligned} mFAAWA_\gamma (\eta _1,\eta _2,\eta _3) =&\sum _{k=1}^{3}\left( \gamma _k\eta _k\right) ,\nonumber \\ =&\left( 1-\dfrac{1}{e^{\{\sum _{k=1}^{n}\gamma _k(-ln(1-P_1\eta _k))^p\}^{1/p}}},\ldots ,1-\dfrac{1}{e^{\{\sum _{k=1}^{n}\gamma _k(-ln(1-P_m\eta _k))^p\}^{1/p}}}\right) ,\nonumber \\ =&\Big (1-\dfrac{1}{e^{\{\gamma _1(-ln(1-P_1\eta _1))^p+\gamma _2(-ln(1-P_1\eta _2))^p+\gamma _3(-ln(1-P_1\eta _3))^p\}^{1/p}}},\nonumber \\&~~~~~~1-\dfrac{1}{e^{\{\gamma _1(-ln(1-P_2\eta _1))^p+\gamma _2(-ln(1-P_2\eta _2))^p+\gamma _3(-ln(1-P_2\eta _3))^p\}^{1/p}}}, \nonumber \\ {}&~~~~~~1-\dfrac{1}{e^{\{\gamma _1(-ln(1-P_3\eta _1))^p+\gamma _2(-ln(1-P_3\eta _2))^p+\gamma _3(-ln(1-P_3\eta _3))^p\}^{1/p}}}\Big ), \end{aligned}$$

For $$p=5$$,

$$\begin{aligned} mFAAWA_\gamma (\eta _1,\eta _2,\eta _3)=&\Big (1-\dfrac{1}{e^{\{0.25(-ln(1-0.52))^5+0.45(-ln(1-0.31))^5+0.30(-ln(1-0.73))^5\}^{1/5}}},\\\\&~~~~1-\dfrac{1}{e^{\{0.25(-ln(1-0.16))^5+0.45(-ln(1-0.80))^5+0.30(-ln(1-0.91))^5\}^{1/5}}}, \\\\ {}&~~~~1-\dfrac{1}{e^{\{0.25(-ln(1-0.37))^5+0.45(-ln(1-0.25))^5+0.30(-ln(1-0.16))^5\}^{1/5}}}\Big ),\\\\ =&(0.6090,0.8596,0.3036). \end{aligned}$$

#### Theorem 3.2

(Idempotent law). Let $\eta _k=\left( P_1\circ \eta _k,P_2\circ \eta _k,\ldots ,P_m\circ \eta _k\right)$ be a collection of ‘*n*’ *m*F numbers with $\eta _k=\eta$ and let $\gamma$ be the weight vector. Then

$$\begin{aligned} mFAAWA_\gamma \left( \eta _1,\eta _2,\ldots ,\eta _n\right) =\eta \end{aligned}$$.

#### Proof

Let $\eta _k=\left( P_1\circ \eta _k,P_2\circ \eta _k,\ldots ,P_m\circ \eta _k\right)$ with $\eta _k=\eta$ for $k=1,2,3,\ldots ,n$, then by Equation (1),

$$\begin{aligned} mFAAWA_\gamma \left( \eta _1,\eta _2,\ldots ,\eta _n\right) =&\sum _{k=1}^{n}\left( \gamma _k\eta _k\right) ,\\ =&\Big (1-\dfrac{1}{e^{\{\sum _{k=1}^{n}\gamma _k(-ln(1-P_1\eta _k))^p\}^{1/p}}},\ldots ,\\ {}&~~1-\dfrac{1}{e^{\{\sum _{k=1}^{n}\gamma _k(-ln(1-P_m\eta _k))^p\}^{1/p}}}\Big ),\\ =&\left( 1-\dfrac{1}{e^{\{(-ln(1-P_1\eta ))^p\}^{1/p}}},\ldots ,1-\dfrac{1}{e^{\{(-ln(1-P_m\eta ))^p\}^{1/p}}}\right) ,\\ =&\left( P_1\circ \eta ,P_2\circ \eta ,\ldots ,P_m\circ \eta \right) ,\\ =&\eta \end{aligned}$$

Thus, $mFAAWA_\gamma \left( \eta _1,\eta _2,\ldots ,\eta _n\right) =\eta$ when $\eta _k=\eta$ with $k=1,2,\ldots , n$. $\square$

Proofs of the following two theorems are similar to the proof of Theorem 3.2, and therefore omitted.

#### Theorem 3.3

(Monotonic Law) Let there be two sets of *m*F numbers $\eta _k$ and $\hat{\eta _k}$ where $k=1,2,\ldots ,n$ with $\eta _k\le \hat{\eta _k}$, then

3 $$\begin{aligned} mFAAWA_\gamma \left( \eta _1,\eta _2,\ldots ,\eta _n\right) \le mFAAWA_\gamma \left( \hat{\eta _1},\hat{\eta _2},\ldots ,\hat{\eta _n}\right) . \end{aligned}$$

#### Theorem 3.4

(Bounded Law) Let $\eta _k=\left( P_1\circ \eta _k,P_2\circ \eta _k,\ldots ,P_m\circ \eta _k\right)$ be the collection of *m*F numbers for $k=1,2,\ldots ,n$ and $\gamma$ represents the weight vector, then

$$\begin{aligned} \eta ^-\le mFAAWA_\gamma \left( \eta _1,\eta _2,\ldots ,\eta _n\right) \le \eta ^+ \end{aligned}$$

where $$\eta ^-$$=$$\min \{{\eta _k}\}$$ and $$\eta ^+$$=$$\max \{{\eta _k}\}$$.

The following defines the *m*FAAOWA AOs.

#### Definition 3.2

Let $\eta _k=\left( P_1\circ \eta _k,P_2\circ \eta _k,\ldots ,P_m\circ \eta _k\right)$ $(1\le k\le n)$ be a collection of *m*F numbers. The *m*FAAOWA AO given by *m*FAAOWA:$\eta ^n\rightarrow \eta$ is defined as:

$$\begin{aligned} mFAAOWA_\gamma \left( \eta _1,\eta _2,\ldots ,\eta _n\right) =\sum _{k=1}^{n}\left( \gamma _k\eta _{\tau (k)}\right) . \end{aligned}$$

Here $\gamma =(\gamma _1,\gamma _2,\ldots ,\gamma _n)$ represents the weight vector and $\tau (k)$ denotes the permutation with $\gamma _{\tau (k-1)}\ge \gamma _{\tau (k)}$.

#### Theorem 3.5

Let $\eta _k=\left( P_1\circ \eta _k,P_2\circ \eta _k,\ldots ,P_m\circ \eta _k\right)$ be the collection of ‘*n*’ *m*F numbers where $k=1,2,\ldots ,n$. The aggregation of these *m*F numbers by *m*FAAOWA AO is given as:

4 $$\begin{aligned} mFAAOWA_\gamma \left( \eta _1,\eta _2,\ldots ,\eta _n\right)&=\sum _{k=1}^{n}\left( \gamma _k\eta _{\tau (k)}\right) ,\nonumber \\&=\Big (1-\dfrac{1}{e^{\{\sum _{k=1}^{n}\gamma _k(-ln(1-P_1\eta _{\tau (k)}))^p\}^{1/p}}},\ldots ,\nonumber \\&~~~~~~1-\dfrac{1}{e^{\{\sum _{k=1}^{n}\gamma _k(-ln(1-P_m\eta _{\tau (k)}))^p\}^{1/p}}}\Big ). \end{aligned}$$

#### Proof

It follows directly from the proof of Theorem 3.1. $\square$

#### Example 3.2

Let $\eta _1=\left( 0.53,0.17,0.71\right)$, $\eta _2=\left( 0.36,0.25,0.41\right)$ and $\eta _3=\left( 0.80,0.18,0.79\right)$ be 3F numbers with weights $\gamma =(0.4,0.5,0.1)$. For $p=3$, the score values are computed as follows:

$$\begin{aligned} S(\eta _1)= & {} \dfrac{0.53+0.17+0.71}{3}=0.4700, \\ S(\eta _2)= & {} \dfrac{0.36+0.25+0.41}{3}=0.3400, \\ S(\eta _3)= & {} \dfrac{0.80+0.18+0.79}{3}= 0.5900. \end{aligned}$$

Clearly $$S(\eta _3)>S(\eta _1)>S(\eta _2)$$. Thus,

$$\begin{aligned} \eta _{\tau (1)}= & {} \eta _3=(0.80,0.18,0.79), \\ \eta _{\tau (2)}= & {} \eta _1=(0.53,0.17,0.71), \\ \eta _{\tau (3)}= & {} \eta _2=(0.36,0.25,0.41). \end{aligned}$$

By using Eq. (4),

$$\begin{aligned} mFAAOWA_\gamma \left( \eta _1,\eta _2,\eta _3\right)&=\Big (1-\dfrac{1}{e^{\{\sum _{k=1}^{n}\gamma _k(-ln(1-P_1\eta _{\tau (k)}))^p\}^{1/p}}},\ldots , 1-\dfrac{1}{e^{\{\sum _{k=1}^{n}\gamma _k(-ln(1-P_m\eta _{\tau (k)}))^p\}^{1/p}}}\Big ),\\&=\Big (1-\dfrac{1}{e^{\{\gamma _1(-ln(1-P_1\eta _1))^p+\gamma _2(-ln(1-P_1\eta _2))^p+\gamma _3(-ln(1-P_1\eta _3))^p\}^{1/p}}},\\&~~~~1-\dfrac{1}{e^{\{\gamma _1(-ln(1-P_2\eta _1))^p+\gamma _2(-ln(1-P_2\eta _2))^p+\gamma _3(-ln(1-P_2\eta _3))^p\}^{1/p}}},\\&~~~~~1-\dfrac{1}{e^{\{\gamma _1(-ln(1-P_3\eta _1))^p+\gamma _2(-ln(1-P_3\eta _2))^p+\gamma _3(-ln(1-P_3\eta _3))^p\}^{1/p}}}\Big ),\\&=\Big (1-\dfrac{1}{e^{\{0.25(-ln(1-0.80))^3+0.30(-ln(1-0.53))^3+0.45(-ln(1-0.36))^3\}^{1/3}}},\\&~~~~~~1-\dfrac{1}{e^{\{0.25(-ln(1-0.18))^3+0.30(-ln(1-0.17))^3+0.45(-ln(1-0.25))^3\}^{1/3}}},\\&~~~~~~1-\dfrac{1}{e^{\{0.25(-ln(1-0.79))^3+0.30(-ln(1-0.71))^3+0.45(-ln(1-0.41))^3\}^{1/3}}}\Big ),\\&=(0.6556,0.2169,0.6884). \end{aligned}$$

#### Remark 3.1

The *m*FAAOWA AOs satisfy all the basic properties including idempotency, monotonicity, and boundedness as discussed in Theorems 3.2, 3.3, and 3.4, respectively.

#### Theorem 3.6

(Commutative Law) Let $\eta _k$ and $\hat{\eta _k}$ be any two families of *m*F numbers where $k=1,2,3,\ldots ,n$. Then

$$\begin{aligned} mFAAOWA_\gamma \left( \eta _1,\eta _2,\ldots ,\eta _n\right) = mFAAOWA_\gamma \left( \hat{\eta _1},\hat{\eta _2},\ldots ,\hat{\eta _n}\right) \end{aligned}$$

where $\hat{\eta _k}$ is a random permutation of $\eta _k$.

#### Proof

It is immediately shown by Definition 3.2. $\square$

Coming definition gives the concept of *m*FAAHWA AOs as hybridization of the previous two AOs.

#### Definition 3.3

Let $\eta _k=\left( P_1\circ \eta _k,P_2\circ \eta _k,\ldots ,P_m\circ \eta _k\right)$ be the collection of *m*F numbers with $k=1,2,\ldots ,n$. Then a mapping *m*FAAHWA:$\eta ^n\rightarrow \eta$ defines the *m*FAAHWA AO as:

$$\begin{aligned} mFAAHWA_\gamma ,_\omega \left( \eta _1,\eta _2,\ldots ,\eta _n\right) =\sum _{k=1}^{n}\left( \gamma _k\hat{\eta }_{\tau (k)}\right) \end{aligned}$$

Here $\gamma$=($\gamma _1,\gamma _2,\ldots ,\gamma _n)$ represents the ordered weights with $\gamma _k\in (0,1]$ and $\sum _{k=1}^{n}\gamma _k=1$. Moreover, $\hat{\eta }_{\tau (k)}$ is the $k-$th biggest weighted *m*F number defined as $\hat{\eta }_{\tau (k)}={n\omega _k}(\eta _k),$ where $\omega =(\omega _1,\omega _2,\ldots ,\omega _n)$ represents an unordered weight vector with $\sum _{k=1}^{n}\omega _k=1$ and $\omega _k\in (0,1]$.

#### Theorem 3.7

Let $\eta _k=\left( P_1\circ \eta _k,P_2\circ \eta _k,\ldots ,P_m\circ \eta _k\right)$ be the collection of ‘*n*’ *m*F numbers where $k=1,2,\ldots ,n$. The aggregation of these *m*F numbers by *m*FAAHWA AO is given as:

$$\begin{aligned} mFAAHWA_\gamma ,_\omega \left( \eta _1,\eta _2,\ldots ,\eta _n\right)&=\sum _{k=1}^{n}\left( \gamma _k\hat{\eta }_{\tau (k)}\right) ,\\&=\Big (1-\dfrac{1}{e^{\{\sum _{k=1}^{n}\gamma _k(-ln(1-P_1\hat{\eta }_{\tau (k)}))^p\}^{1/p}}},\ldots ,\\ {}&~~~~~~1-\dfrac{1}{e^{\{\sum _{k=1}^{n}\gamma _k(-ln(1-P_m\hat{\eta }_{\tau (k)}))^p\}^{1/p}}}\Big ). \end{aligned}$$

#### Example 3.3

Let $\eta _1$=$\left( 0.84,0.33,0.51\right)$, $\eta _2$=$\left( 0.27,0.63,0.16\right)$, and $\eta _3=\left( 0.62,0.14,0.70\right)$ be three 3F numbers with weight vectors $\gamma =(0.25,0.30,0.45)$ and $\omega =(0.35,0.50,0.15)$. Then by Definition 3.3, for $p=3$,

$$\begin{aligned} \hat{\eta _1}=&\Big (1-\dfrac{1}{e^{\{n\omega _1(-ln(1-P_1\eta _1))^p\}^{1/p}}},1-\dfrac{1}{e^{\{n\omega _1(-ln(1-P_2\eta _1))^p\}^{1/p}}},1-\dfrac{1}{e^{\{n\omega _1(-ln(1-P_3\eta _1))^p\}^{1/p}}}\Big )\\ =&\Big (1-\dfrac{1}{e^{\{3\times 0.25(-ln(1-0.84))^3\}^{1/3}}},1-\dfrac{1}{e^{\{3\times 0.25(-ln(1-0.33))^3\}^{1/3}}},1-\dfrac{1}{e^{\{3\times 0.25(-ln(1-0.51))^3\}^{1/3}}}\Big )\\ =&\Big (0.8108,0.3050,0.4770\Big ) \end{aligned}$$

Similarly,

$$\begin{aligned} \hat{\eta _2}&=\Big (1-\dfrac{1}{e^{\{3\times 0.30(-ln(1-0.27))^3\}^{1/3}}},1-\dfrac{1}{e^{\{3\times 0.30(-ln(1-0.63))^3\}^{1/3}}},1-\dfrac{1}{e^{\{3\times 0.30(-ln(1-0.16))^3\}^{1/3}}}\Big )\\&=\Big (0.2620,0.6171,0.1549\Big )\\ \hat{\eta _3}&=\Big (1-\dfrac{1}{e^{\{3\times 0.45(-ln(1-0.62))^3\}^{1/3}}},1-\dfrac{1}{e^{\{3\times 0.45(-ln(1-0.14))^3\}^{1/3}}},1-\dfrac{1}{e^{\{3\times 0.45(-ln(1-0.70))^3\}^{1/3}}}\Big )\\&=\Big (0.6568,0.1535,0.7357\Big ) \end{aligned}$$

The score values for these computed *m*F numbers are calculated as:

$$\begin{aligned} S(\hat{\eta _1})= & {} \dfrac{0.8108+0.3050+0.4770}{3}=0.5309, \\ S(\hat{\eta _2})= & {} \dfrac{0.2620+0.6171+0.1549}{3}=0.3447, \\ S(\hat{\eta _3})= & {} \dfrac{0.6568+0.1535+0.7357}{3}=0.5153. \end{aligned}$$

Since $$S(\hat{\eta _1})>S(\hat{\eta _3})>S(\hat{\eta _2}),$$ therefore

$$\begin{aligned} \hat{\eta }_{\tau (1)}= & {} \hat{\eta _1}=(0.8108,0.3050,0.4770), \\ \hat{\eta }_{\tau (2)}= & {} \hat{\eta _3}=(0.6568,0.1535,0.7357), \\ \hat{\eta }_{\tau (3)}= & {} \hat{\eta _2}=(0.2620,0.6171,0.1549). \end{aligned}$$

Finally, by applying Definition 3.3 for $p=3$,

$$\begin{aligned} mFAAHWA_\gamma ,_\omega \left( \eta _1,\eta _2,\ldots ,\eta _n\right)&=\sum _{k=1}^{n}\left( \gamma _k\hat{\eta }_{\tau (k)}\right) \\&=\Big (1-\dfrac{1}{e^{\{\sum _{k=1}^{3}\gamma _k(-ln(1-P_1\hat{\eta }_{\tau (k)}))^p\}^{1/p}}},\ldots ,\\ {}&~~~~~ 1-\dfrac{1}{e^{\{\sum _{k=1}^{3}\gamma _k(-ln(1-P_3\hat{\eta }_{\tau (k)}))^p\}^{1/p}}}\Big )\\&=\Big (1-\dfrac{1}{e^{\{0.35(-ln(1-0.8108))^3+0.50(-ln(1-0.6568))^3+0.15(-ln(1-0.2620))^3\}^{1/3}}},\\&~~~~ 1-\dfrac{1}{e^{\{0.35(-ln(1-0.3050))^3+0.50(-ln(1-0.1535))^3+0.15(-ln(1-0.6171))^3\}^{1/3}}},\\&~~~~ 1-\dfrac{1}{e^{\{0.35(-ln(1-0.4770))^3+0.50(-ln(1-0.7357))^3+15(-ln(1-0.1549))^3\}^{1/3}}}\Big )\\&=\big (0.7293,0.4135,0.6618\big ) \end{aligned}$$

### *m*F Aczel–Alsina weighted geometric AOs

This subsection presents *m*F Aczel–Alsina weighted geometric AOs and their properties.

#### Definition 3.4

Let $\eta _k=\left( P_1\circ \eta _k,P_2\circ \eta _k,\ldots ,P_m\circ \eta _k\right)$ be the set of *m*F numbers where $k=1,2,\ldots ,n$, then a mapping *m*FAAWG:$\eta ^n\rightarrow \eta$ defines the *m*FAAWG AO given as:

$$\begin{aligned} mFAAWG_\gamma \left( \eta _1,\eta _2,\ldots ,\eta _n\right) =\bigotimes _{k=1}^{n}\left( \eta _k\right) ^{\gamma _k} \end{aligned}$$

where $\gamma$=($\gamma _1,\gamma _2,\ldots ,\gamma _n)$ is the weight vector satisfying $\sum _{k=1}^{n}=1$ for $\gamma _k\in (0,1]$.

#### Theorem 3.8

Let $\eta _k=\left( P_1\circ \eta _k,P_2\circ \eta _k,\ldots ,P_m\circ \eta _k\right)$ be the collection of *m*F numbers. then the output obtained by aggregating these *m*FNs using *m*FAAWG AO is again an *m*F number. Mathematically,

$$\begin{aligned} mFAAWG_\gamma \left( \eta _1,\eta _2,\ldots ,\eta _n\right)&=\bigotimes _{k=1}^{n}\left( \eta _k\right) ^{\gamma _k},\\&=\Big (\dfrac{1}{e^{\{\sum _{k=1}^{n}\gamma _k(-lnP_1\eta _k)^p\}^{1/p}}},\ldots ,\dfrac{1}{e^{\{\sum _{k=1}^{n}\gamma _k(-lnP_1\eta _k)^p\}^{1/p}}}\Big ). \end{aligned}$$

#### Proof

Its proof is same as of Theorem 3.1. $\square$

#### Example 3.4

Let $\eta _1=(0.64,0.13,0.46), \eta _2=(0.78,0.32,0.98)$ and $\eta _3=(0.82,0.51,0.32)$ be 3F numbers with weights $\gamma =(0.25,0.45,0.30)$. Then for $p=3$,

$$\begin{aligned} mFAAWG_\gamma \left( \eta _1,\eta _2,\ldots ,\eta _n\right)&=\bigotimes _{k=1}^{n}\left( \eta _k\right) ^{\gamma _k},\\&=\Big (\dfrac{1}{e^{\{\sum _{k=1}^{n}\gamma _k(-lnP_1\eta _k)^p\}^{1/p}}},\ldots ,\dfrac{1}{e^{\{\sum _{k=1}^{n}\gamma _k(-lnP_1\eta _k)^p\}^{1/p}}}\Big ),\\&=\Big (\dfrac{1}{e^{\{\gamma _1(-lnP_1\eta _1)^p+\gamma _2(-lnP_1\eta _2)^p+\gamma _3(-lnP_1\eta _3)^p\}^{1/p}}},\\&~~~~~~\dfrac{1}{e^{\{\gamma _1(-lnP_2\eta _1)^p+\gamma _2(-lnP_2\eta _2)^p+\gamma _3(-lnP_2\eta _3)^p\}^{1/p}}},\\&~~~~~~\dfrac{1}{e^{\{\gamma _1(-lnP_3\eta _1)^p+\gamma _2(-lnP_3\eta _2)^p+\gamma _3(-lnP_3\eta _3)^p\}^{1/p}}}\Big ),\\&=\Big (\dfrac{1}{e^{\{0.25(-ln0.64)^3+0.45(-ln0.78)^3+0.30(-ln0.82)^3\}^{1/3}}},\\&~~~~~~\dfrac{1}{e^{\{0.25(-ln(0.13))^3+0.45(-ln(0.32))^3+0.30(-ln(0.51))^3\}^{1/3}}},\\&~~~~~~\dfrac{1}{e^{\{0.25(-ln(0.46))^3+0.45(-ln(0.98))^3+0.30(-ln(0.32))^3\}^{1/3}}}\Big ),\\&=\left( 0.7293,0.2410,0.4384\right) . \end{aligned}$$

#### Theorem 3.9

(Idempotent Law) Let $\eta _k=\left( P_1\circ \eta _k,P_2\circ \eta _k,\ldots ,P_m\circ \eta _k\right)$ be the family of *m*F numbers where $k=1,2,3,\ldots ,n$ such that $\eta _k=\eta$ and $\gamma$ contains the corresponding weights, then

$$\begin{aligned} mFAAWG_\gamma \left( \eta _1,\eta _2,\ldots ,\eta _n\right) =\eta . \end{aligned}$$

#### Theorem 3.10

(Monotonic Law) Let $\eta _k=\left( P_1\circ \eta _k,\ldots ,P_m\circ \eta _k\right)$ and $\hat{\eta _k}=\left( P_1\circ \hat{\eta _k},\ldots ,P_m\circ \hat{\eta _k}\right)$ be two sets of *m*F numbers and $\gamma$ consists of the corresponding weights, if $\eta _k\le \hat{\eta _k}$ where *k* varies from 1 to *n*, then

$$\begin{aligned} mFAAWG_\gamma \left( \eta _1,\eta _2,\ldots ,\eta _n\right) \le mFAAWG_\gamma \left( \hat{\eta _1},\hat{\eta _2},\ldots ,\hat{\eta _n}\right) . \end{aligned}$$

#### Theorem 3.11

(Bounded Law) Let $\eta _k=\left( P_1\circ \eta _k,\ldots ,P_m\circ \eta _k\right)$ and $\hat{\eta _k}=\left( P_1\circ \hat{\eta _k},\ldots ,P_m\circ \hat{\eta _k}\right)$ be two sets of *m*F numbers, and $\gamma$ contains the respective weights. Then

$$\begin{aligned} \eta ^-\le mFAAWG_\gamma \left( \eta _1,\eta _2,\ldots ,\eta _n\right) \le \eta ^+ \end{aligned}$$

where $$\eta ^-=\min {\eta _k}$$ and $$\eta ^+=\max {\eta _k}$$.

Next definition gives the notion of *m*FAAOWG AOs.

#### Definition 3.5

Let $\eta _k=\left( P_1\circ \eta _k,P_2\circ \eta _k,\ldots ,P_m\circ \eta _k\right)$ $(1\le k\le n)$ be a collection of *m*F numbers. The *m*FAAOWG AO given by *m*FAAOWG:$\eta ^n\rightarrow \eta$ is defined as:

$$\begin{aligned} mFAAOWG_\gamma \left( \eta _1,\eta _2,\ldots ,\eta _n\right) =\bigotimes _{k=1}^{n}\left( \eta _{\tau (k)}\right) ^{\gamma _k}. \end{aligned}$$

Here $\gamma =(\gamma _1,\gamma _2,\ldots ,\gamma _n)$ represents the weight vector and $\tau (k)$ denotes the permutation with $\gamma _{\tau (k-1)}\ge \gamma _{\tau (k)}$.

#### Theorem 3.12

Let $\eta _k=\left( P_1\circ \eta _k,\ldots ,P_m\circ \eta _k\right)$ be the set of *m*F numbers with $k=1,2,\ldots ,n$. Then, an output value obtained after aggregation of these numbers using the *m*FAAOWG is also an *m*FNs given by

$$\begin{aligned} mFAAOWG_\gamma \left( \eta _1,\eta _2,\ldots ,\eta _n\right)&=\bigotimes _{k=1}^{n}\left( \eta _{\tau (k)}\right) ^{\gamma _k},\\&=\Big (\dfrac{1}{e^{\{\sum _{k=1}^{n}\gamma _k(-lnP_1\eta _{\tau (k)})^p\}^{1/p}}},\ldots ,\dfrac{1}{e^{\{\sum _{k=1}^{n}\gamma _k(-lnP_m\eta _{\tau (k)})^p\}^{1/p}}}\Big ). \end{aligned}$$

#### Example 3.5

Let $\eta _1=(0.43,0.71,0.28), ~\eta _2=(0.32,0.63,0.19)$, and $\eta _3=(0.25,0.90,0.53)$ be three 3F numbers with weight vector $\gamma =(0.25,0.30,0.45)$. The score values of these *m*F numbers are calculated as below:

$$\begin{aligned} S(\hat{\eta _1})= & {} \dfrac{0.43+0.71+0.28}{3}=0.4733, \\ S(\hat{\eta _2})= & {} \dfrac{0.32+0.63+0.19}{3}=0.3800, \\ S(\hat{\eta _3})= & {} \dfrac{0.25+0.90+0.53}{3}=0.5600. \end{aligned}$$

This implies $S(\hat{\eta _3})>S(\hat{\eta _1})>S(\hat{\eta _2})$. Therefore,

$$\begin{aligned} \eta _{\tau (1)}= & {} \hat{\eta _3}=(0.25,0.90,0.53), \\ \eta _{\tau (2)}= & {} \hat{\eta _1}=(0.43,0.71,0.28), \\ \eta _{\tau (3)}= & {} \hat{\eta _2}=(0.32,0.63,0.19). \end{aligned}$$

Now for $p=3$, by applying Definition 3.5,

$$\begin{aligned} mFAAOWG_\gamma \left( \eta _1,\eta _2,\eta _3\right)&=\bigotimes _{k=1}^{3}\left( \eta _{\tau (k)}\right) ^{\gamma _k},\\&=\Big (\dfrac{1}{e^{\{\sum _{k=1}^{n}\gamma _k(-lnP_1\eta _{\tau (k)})^p\}^{1/p}}},\ldots ,\dfrac{1}{e^{\{\sum _{k=1}^{n}\gamma _k(-lnP_3\eta _{\tau (k)})^p\}^{1/p}}}\Big ),\\&=\Big (\dfrac{1}{e^{\{0.25(-ln0.25)^3+0.30(-ln0.43)^3+0.45(-ln0.32)^3\}^{1/3}}},\\&~~~~~~\dfrac{1}{e^{\{0.25(-ln(0.90))^3+0.30(-ln(0.71))^3+0.45(-ln(0.63))^3\}^{1/3}}},\\ {}&~~~~~~\dfrac{1}{e^{\{0.25(-ln(0.53))^3+0.30(-ln(0.28))^3+0.45(-ln(0.19))^3\}^{1/3}}}\Big ),\\&=\left( 0.3173,0.6810,0.2466\right) . \end{aligned}$$

#### Remark 3.2

The *m*FAAOWG operators satisfy the fundamental properties including idempotency, monotonocity and boundedness as studied in Theorems 3.2, 3.3, and 3.4.

#### Theorem 3.13

(Commutative Law) Let $\eta _k$ and $\hat{\eta _k}$ be any two collections of *m*F numbers where $k=1,2,\ldots ,n$, then

$$\begin{aligned} mFAAOWG_\gamma \left( \eta _1,\eta _2,\ldots ,\eta _n\right) = mFAAOWG_\gamma \left( \hat{\eta _1},\hat{\eta _2},\ldots ,\hat{\eta _n}\right) \end{aligned}$$

Here $\hat{\eta _k}$ is any permutation of $\eta _k$.

#### Proof

It is immediately followed by Definition 3.5$\square$

Next definition combines the features of *m*FAAWG and *m*FAAOWG AOs to form *m*FAAHWG AOs.

#### Definition 3.6

Let $\eta _k=\left( P_1\circ \eta _k,P_2\circ \eta _k,\ldots ,P_m\circ \eta _k\right)$ be the collection of *m*F numbers with $k=1,2,\ldots ,n$. Then a mapping *m*FAAHWG:$\eta ^n \rightarrow \eta$ defines the *m*FAAHWG AO as:

$$\begin{aligned} mFAAHWG_{\gamma ,\omega }\left( \eta _1,\eta _2,\ldots ,\eta _n\right) =\bigotimes _{k=1}^{n}\left( \hat{\eta }_{\tau (k)}\right) ^{\gamma _k} \end{aligned}$$

Here $\gamma =(\gamma _1,\gamma _2,\ldots ,\gamma _n)$ represents the ordered weights with $\gamma _k\in (0,1]$ and $\sum _{k=1}^{n}\gamma _k=1$. Moreover, $\hat{\eta }_{\tau (k)}$ is the $k$-th biggest weighted *m*F number defined as $\hat{\eta }_{\tau (k)}=(\eta _k)^{n\omega _k},$ where $\omega =(\omega _1,\omega _2,\ldots ,\omega _n)$ represents an unordered weight vector with $\sum _{k=1}^{n}\omega _k=1$ and $\omega _k\in (0,1]$.

#### Theorem 3.14

Let $\eta _k=\left( P_1\circ \eta _k,P_2\circ \eta _k,\ldots ,P_m\circ \eta _k\right)$ be the family of *m*F numbers with $k=1,2,\ldots ,n$, then the aggregated value obtained after aggregation of these numbers using the *m*FAAHWG operator is also an *m*F number given by

$$\begin{aligned} mFAAHWG_{\gamma ,\omega }\left( \eta _1,\eta _2,\ldots ,\eta _n\right) =\bigotimes _{k=1}^{n}\left( \hat{\eta }_{\tau (k)}\right) ^{\gamma _k}. \end{aligned}$$

#### Example 3.6

Let $\eta _1=(0.35,0.13,0.55),\eta _2=(0.17,0.63,0.28)$ and $\eta _3=(0.54,0.21,0.48)$ be three 3F numbers, and $\gamma =(0.30,0.25,0.45)$ be an associated weight-vector, then

$$\begin{aligned} \hat{\eta _1}&=\Big (\dfrac{1}{e^{\{n\omega _1(-lnP_1\eta _1)^p\}^{1/p}}},\dfrac{1}{e^{\{n\omega _1(-lnP_2\eta _1)^p\}^{1/p}}},\dfrac{1}{e^{\{n\omega _1(-lnP_3\eta _1)^p\}^{1/p}}}\Big )\\&=\Big (\dfrac{1}{e^{\{3\times 0.30(-ln0.35)^3\}^{1/3}}},\dfrac{1}{e^{\{3\times 0.30(-ln0.13)^3\}^{1/3}}},\dfrac{1}{e^{\{3\times 0.30(-ln0.55)^3\}^{1/3}}}\Big )\\&=(0.3127,0.1395,0.5615)\\ \textrm{Similarly,}\\ \hat{\eta _2}&=\Big (\dfrac{1}{e^{\{3\times 0.25(-ln0.17)^3\}^{1/3}}},\dfrac{1}{e^{\{3\times 0.25(-ln0.63)^3\}^{1/3}}},\dfrac{1}{e^{\{3\times 0.25(-ln0.28)^3\}^{1/3}}}\Big )\\\\&=(0.1999,0.6572,0.3146)\\\\ \hat{\eta _3}&=\Big (\dfrac{1}{e^{\{3\times 0.45(-ln0.54)^3\}^{1/3}}},\dfrac{1}{e^{\{3\times 0.45(-ln0.21)^3\}^{1/3}}},\dfrac{1}{e^{\{3\times 0.45(-ln0.48)^3\}^{1/3}}}\Big )\\\\&=(0.5061,0.1782,0.4443)\\ \end{aligned}$$

Corresponding score values are calculated as:

$$\begin{aligned} S(\hat{\eta _1})&=\dfrac{0.3127+0.1395+0.5615}{3} =0.3379,\\ S(\hat{\eta _2})&=\dfrac{0.1999+0.6572+0.3146}{3} =0.3906,\\ S(\hat{\eta _3})&=\dfrac{0.5061+0.1782+0.4443}{3} =0.3762. \end{aligned}$$

Since $$S(\hat{\eta _2})> S(\hat{\eta _3})>S(\hat{\eta _1})$$, therefore

$$\begin{aligned} \eta _{\tau (1)}= & {} \hat{\eta _2}=(0.1999,0.6572,0.3146), \\ \eta _{\tau (2)}= & {} \hat{\eta _3}=(0.5061,0.1782,0.4443), \\ \eta _{\tau (3)}= & {} \hat{\eta _1}=(0.3127,0.1395,0.5615). \end{aligned}$$

By using Definition 3.6 and fixing $p=3$,

$$\begin{aligned} mFAAHWG_{\gamma ,\omega }\left( \eta _1,\eta _2,\eta _3\right)&=\bigotimes _{k=1}^{3}\left( \hat{\eta }_{\tau (k)}\right) ^{\gamma _k},\\&=\Big (\dfrac{1}{e^{\{\sum _{k=1}^{3}\gamma _k(-lnP_1\eta _{\tau (k)})^p\}^{1/p}}},\ldots ,\dfrac{1}{e^{\{\sum _{k=1}^{3}\gamma _k(-lnP_3\eta _{\tau (k)})^p\}^{1/p}}}\Big ),\\&=\Big (\dfrac{1}{e^{\{0.25(-ln0.1999)^3+0.15(-ln0.5061)^3+0.60(-ln0.3127)^3\}^{1/3}}},\\&~~~~~~\dfrac{1}{e^{\{0.25(-ln(0.6572))^3+0.15(-ln(0.1782))^3+0.60(-ln(0.1395))^3\}^{1/3}}},\\ {}&~~~~~~\dfrac{1}{e^{\{0.25(-ln(0.3146))^3+0.15(-ln(0.4443))^3+0.60(-ln(0.5615))^3\}^{1/3}}}\Big ),\\&=(0.2817,0.1735,0.4339). \end{aligned}$$

## Applications to MCDM with *m*F information

This section aims to illustrate the practical implementation of newly proposed AOs. Two multi-polar site selection problems are intricately discussed and solved using newly developed algorithm based on *m*FAAWA and *m*FAAWG AOs.

### Definition 4.1

Consider $\{X_1,X_2,\ldots ,X_n\}$ is the universal set and $\{T_1,T_2,\ldots ,T_k\}$ is the universe of attributes. Suppose $\gamma =(\gamma _1,\gamma _2,\ldots ,\gamma _k)$ is the weight vector corresponding to attributes $T_i~(1\le i\le k)$. Then the *m*F decision-matrix representing the estimations of experts in *m*F environment is formulated as

$$\begin{aligned} \tilde{\mathfrak {N}}=(\tilde{\mathfrak {q}}_{it})_{n\times k}=\big ( P_1\circ \eta _{it},P_2\circ \eta _{it},\ldots ,P_m\circ \eta _{it}\big )_{n\times k} \end{aligned}$$

**Figure 1:**
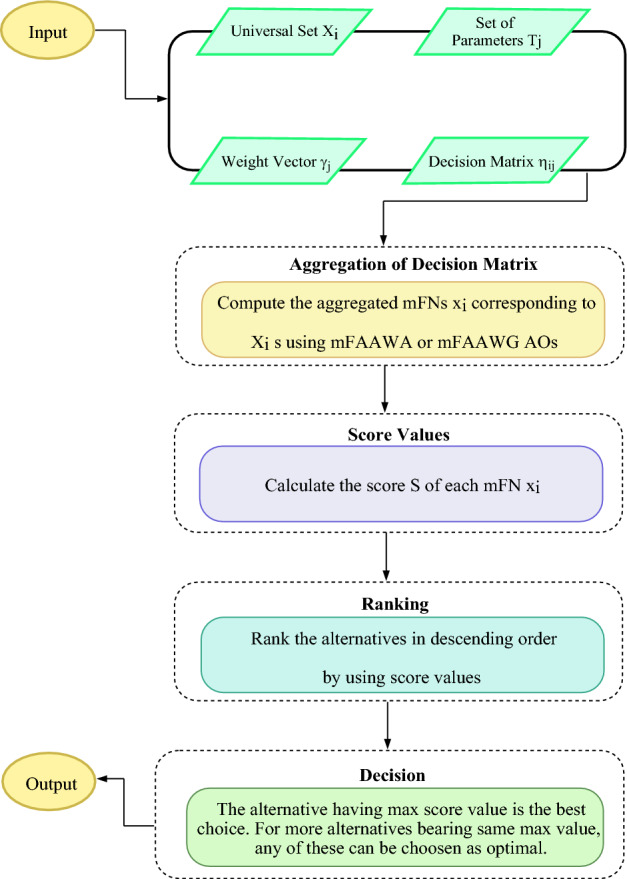
Flowchart of Algorithm 1.

Algorithm 1 represents the proposed method of decision-making with *m*FAAWA and *m*FAAWG AOs. Figure 1 gives a pictorial explanation of the proposed algorithm.

**Figure:**
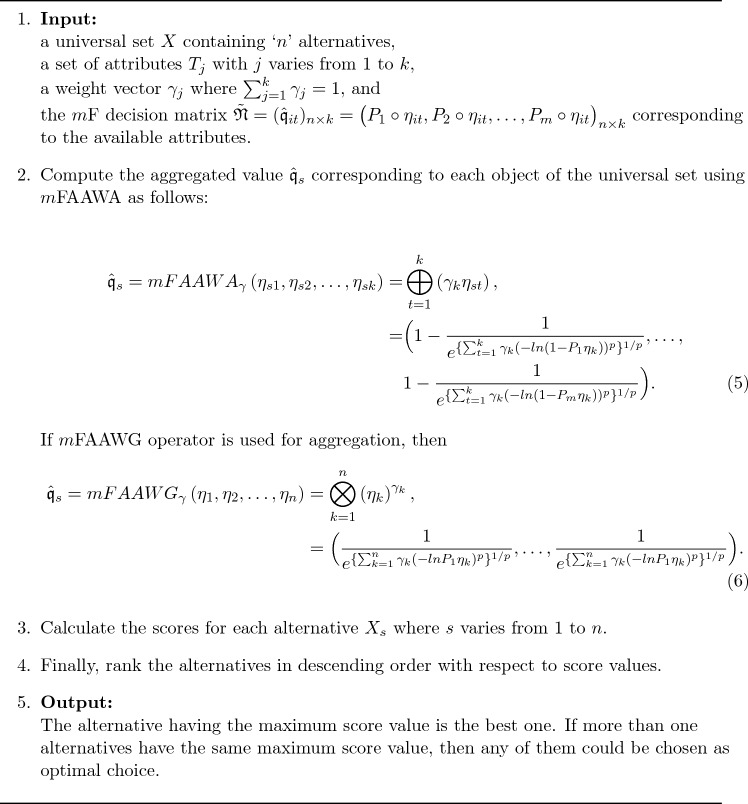
Algorithm 1: Selection of best option using *m*FAAWA or mFAAWG operators.

### Selection of suitable site for desalination plant

The planet earth inhabiting around eight billion people has only 2.5% of its water categorized as fresh water. And unfortunately, only a fraction of this part is usable by the people on earth. The remaining water is sea water (salt water) unuseable in its current state, specifically for drinking. Many parts of the world have no or limited access to the fresh water. Such places make use of the process of desalination (removal of salts from water leaving desalinated water) in desalination plants to turn sea water into drinking and useable water. Globally around 1% of the total drinking water is provided by desalination. This seemingly low percentage accounts to more than 300 million people from around 150 countries of the world, depending partially or completely on this desalinated water for their daily requirements. The island countries and the middle east region heavily depend on desalination. Countries like Maldives, Kuwait, Bahrain, UAE, Saudi Arabia, etc., heavily contribute to the global desalinated water production. Middle east generates around 60% desalinated water of the whole world with only Saudi Arabia producing 117 million cubic feet per day. This heavy contribution accounts for 50% of the fresh water needs of the Saudi Arabian inhabitants. Desalination is an effective solution for the drinking water requirements. However, it is a complicated process with many possible concerns including high power consumption (which may lead to more fossil fuel pollution in case of independent power sources) for pressurization, intake, and reverse osmosis; environmental concerns including possible damage to the marine life, pollution of sea water, non-uniform salinity, heavy brine (brine refers to solution of water containing a lot more salt than the sea water, returned to sea after desalination) disposal, water pre-treatment chemicals disposal, and increased erosion; social and legal concerns including the use of public water, effects to the surrounding inhabitants, effects to legal rights of correspondents, water distribution, possible pollution, employment opportunities, and finally, the financial constraints. Taking these concerns into consideration, the site for a desalination plant must be choosen wisely, as it has long-lasting impacts on the overall production, maintenance, and surrounding areas. Consider a country dependent on sea water is concerned about its increasing need of drinking water. Despite its many coastal areas, the selection of the best site for construction of a desalination plant is crucial and worthy of critical decision-making. A committee including engineers, marine biologists, desalination experts, public representatives, government representatives, approval committee members, geography and topography scientists, local non-governmental organizations, and the consulted environmental standards testing members is constructed. This committee discusses the sites with initial surveys and tests, and comes up with 15 most suitable sites for the desalination plant. For the next phase of detailed evaluation of these sites, four parameters including location, sea-water quality, technical feasibility, and environment friendliness, are choosen by the committee. These four parameters are further categorized into four sub-parameters as described below:

1. Location
  (i) Near a suitable brine discharge area: For minimizing the structure and energy needed to discharge brine back into the sea safely.
  (ii) Near a power supply source or transmission: For eliminating the need of an independent power source and minimizing the power transmission costs.
  (iii) Close to a water supply main conveyor: Minimal distance from the main conveyor ensures safe and cheap transport and administration of the treated water, which can be further distributed by the conveyor easily.
  (iv) Near a well-structured road network: Desalination plant connected to or near a structured road network allows easy transport of machinery and resources during its construction and increases accessibility for further operations.
2. Seawater quality
  (i) Low annual silt density: Low annual silt density range represents lower fouling capacity of sea water in the RO process ensuring longer and reliable operation of the membrane surfaces.
  (ii) Steady and suitable temperature: Steady and suitable temperature of the water ensures health of the reverse osmosis plant.
  (iii) Least contamination risk: Intake away from ports or industries ensuring least contamination risk from the hazardous pollutants.
  (iv) Low turbidity: Low turbidity ensures lower pre-treatment processing power and costs.
3. Technical feasibility
  (i) Intake structures invulnerability against waves and storms: Intake structures should be located and designed so that they have minimal negative affects from the possible sea-waves and storms.
  (ii) Open intake suitability: Suitability for open intake ensures more water production as compared to walled intake.
  (iii) Safe brine discharge and quick dilution: Safe discharge of brine back into the sea water with possible post-processing ensuring quick dilution of the brine is necessary and is dependant on the distance from brine disposal site, height of the plant, post-processing, and dispersal.
  (iv) Ease of water transportation: Topography and behaviour of the site should be helpful in structuring plant for easily transporting the water throughout the process with minimum power.
4. Environment freindliness
  (i) Safe distance from protected areas: Conserved and protected areas like marine ecosystems, wetlands, etc., must be at a safe distance from the desalination plant to protect them from possible intake and brine discharge effects.
  (ii) Distant from inhabited areas: Safe distance from communities and inhabitants must be maintained to reduce the effect of possible noise and smoke pollution.
  (iii) Safety of marine life: The intake, structure, power-source, and byproduct discharge should pose minimum to no harm to any marine life nearby.
  (iv) Safety mitigation against possible erosion: The site selection ensures limitation of possible erosion in order to maintain the seabed topography, and reduce the possible adverse effects.

After analyzing the sites $X_{i}: 1\le i\le 15$ with respect to the parameters $T_j: 1\le j\le 4$, the committee generates a collective report in the form of a 4F decision matrix as shown in Table 1. The committee considers Algorithm 1 (flowchart in Fig. 1) to select the best site from the available most suitable sites. Consequently, committee assigns weights to the parameters as follows:

$$\begin{aligned} \gamma _1=0.25,\quad \gamma _2=0.30,\quad \gamma _3=0.35,\quad \gamma _4=0.10. \end{aligned}$$

**Table 1:** 4F decision matrix for desalination plant sites.

	$T_1$	$T_2$	$T_3$	$T_4$
$M_1$	(0.47, 0.19, 0.31, 0.72)	(0.76, 0.29, 0.32, 0.41)	(0.55, 0.17, 0.68, 0.82)	(0.81, 0.32, 0.11, 0.59)
$M_2$	(0.37, 0.18, 0.60, 0.51)	(0.57, 0.41, 0.77, 0.39)	(0.28, 0.38, 0.13, 0.91)	(0.93, 0.19, 0.82, 0.23)
$M_3$	(0.28, 0.43, 0.15, 0.73)	(0.73, 0.38, 0.50, 0.26)	(0.61, 0.15, 0.89, 0.50)	(0.53, 0.38, 0.42, 0.60)
$M_4$	(0.43, 0.17, 0.29, 0.80)	(0.80, 0.28, 0.67, 0.41)	(0.61, 0.35, 0.48, 0.30)	(0.35, 0.17, 0.49, 0.62)
$M_5$	(0.55, 0.27, 0.60, 0.42)	(0.41, 0.80, 0.11, 0.53)	(0.32, 0.91, 0.15, 0.77)	(0.78, 0.33, 0.50, 0.38)
$M_6$	(0.44, 0.17, 0.66, 0.89)	(0.81, 0.63, 0.41, 0.42)	(0.91, 0.62, 0.15, 0.38)	(0.33, 0.17, 0.64, 0.90)
$M_7$	(0.13, 0.21, 0.47, 0.67)	(0.61, 0.52, 0.17, 0.19)	(0.34, 0.17, 0.60, 0.90)	(0.91, 0.79, 0.82, 0.37)
$M_8$	(0.72, 0.18, 0.59, 0.32)	(0.33, 0.81, 0.26, 0.74)	(0.90, 0.15, 0.52, 0.80)	(0.81, 0.67, 0.42, 0.97)
$M_9$	(0.33, 0.18, 0.83, 0.25)	(0.92, 0.22, 0.63, 0.40)	(0.82, 0.37, 0.18, 0.70)	(0.25, 0.67, 0.10, 0.38)
$M_{10}$	(0.61, 0.33, 0.91, 0.19)	(0.15, 0.27, 0.34, 0.60)	(0.84, 0.65, 0.14, 0.10)	(0.23, 0.40, 0.75, 0.31)
$M_{11}$	(0.85, 0.46, 0.37, 0.68)	(0.11, 0.67, 0.28, 0.81)	(0.35, 0.67, 0.10, 0.99)	(0.53, 0.78, 0.10, 0.35)
$M_{12}$	(0.64, 0.47, 0.25, 0.83)	(0.75, 0.19, 0.47, 0.50)	(0.92, 0.15, 0.73, 0.33)	(0.25, 0.61, 0.47, 0.84)
$M_{13}$	(0.18, 0.41, 0.34, 0.63)	(0.89, 0.43, 0.19, 0.33)	(0.65, 0.30, 0.92, 0.29)	(0.40, 0.83, 0.44, 0.18)
$M_{14}$	(0.57, 0.28, 0.80, 0.33)	(0.85, 0.57, 0.37, 0.97)	(0.48, 0.16, 0.61, 0.87)	(0.19, 0.63, 0.42, 0.91)
$M_{15}$	(0.30, 0.83, 0.67, 0.53)	(0.48, 0.58, 0.99, 0.13)	(0.27, 0.37, 0.49, 0.51)	(0.90, 0.83, 0.71, 0.63)

Firstly, the optimistic approach is carried out using *m*FAAWA aggregation to choose the best site.

Step 1 Let $p=3$. Then using the *m*FAAWA operator, the values $\hat{\mathfrak {q}}_{s}$ for the desalination plant sites $X_s: 1\le s\le 15$ are calculated as:
  $$\begin{aligned} \hat{\mathfrak {q}}_1=(0.6874,0.2456,0.5588,0.7376),&\quad \quad \hat{\mathfrak {q}}_2=(0.7220,0.3560,0.6932,0.8194),\\ \hat{\mathfrak {q}}_3=(0.6342,0.3608,0.7923,0.5990),&\quad \quad \hat{\mathfrak {q}}_4=(0.6892,0.2914,0.5587,0.6537),\\ \hat{\mathfrak {q}}_5=(0.5567,0.8400,0.4570,0.6635),&\quad \quad \hat{\mathfrak {q}}_6=(0.8425,0.5726,0.5395,0.7954),\\ \hat{\mathfrak {q}}_7=(0.6949,0.5488,0.6107,0.8110),&\quad \quad \hat{\mathfrak {q}}_8=(0.8244,0.6828,0.5024,0.8456),\\ \hat{\mathfrak {q}}_9=(0.8470,0.4298,0.6960,0.5827),&\quad \quad \hat{\mathfrak {q}}_{10}=(0.7362,0.5336,0.7894,0.4622),\\ \hat{\mathfrak {q}}_{11}=(0.7023,0.6634,0.2808,0.9631),&\quad \quad \hat{\mathfrak {q}}_{12}=(0.8483,0.4134,0.6184,0.7237),\\ \hat{\mathfrak {q}}_{13}=(0.7854,0.5817,0.8321,0.4801),&\quad \quad \hat{\mathfrak {q}}_{14}=(0.7327,0.4839,0.6716,0.9248),\\ \hat{\mathfrak {q}}_{15}=(0.6668,0.7264,0.9552,0.5003). \end{aligned}$$
Step 2 The score values $S(\hat{\mathfrak {q}}_s)$ of all the above computed 4F numbers $\hat{\mathfrak {q}}_s$ are provided as:
  $$\begin{aligned} S(\hat{\mathfrak {q}}_1)&=0.5574,~\quad S(\hat{\mathfrak {q}}_2)=0.6476,~\quad S(\hat{\mathfrak {q}}_3)=0.5966,~\quad S(\hat{\mathfrak {q}}_4)=0.5482,\\ S(\hat{\mathfrak {q}}_5)&=0.6293,~\quad S(\hat{\mathfrak {q}}_6)=0.6875,~\quad S(\hat{\mathfrak {q}}_7)=0.6663,~\quad S(\hat{\mathfrak {q}}_8)=0.7138,\\ S(\hat{\mathfrak {q}}_9)&=0.6389,\quad S(\hat{\mathfrak {q}}_{10})=0.6304,\quad S(\hat{\mathfrak {q}}_{11})=0.6524,\quad S(\hat{\mathfrak {q}}_{12})=0.6510,\\ S(\hat{\mathfrak {q}}_{13})&=0.6698,\quad S(\hat{\mathfrak {q}}_{14})=0.7033,\quad S(\hat{\mathfrak {q}}_{15})=0.7122. \end{aligned}$$
Step 3 Finally according to the above scores, the objects are ranked as follows:
  $$\begin{aligned} X_8>X_{15}>X_{14}>X_6>X_{13}>X_{7}>X_{11}>X_{12}>X_{2}>X_9>X_{10}>X_5>X_3>X_4>X_1. \end{aligned}$$

Hence, $X_8$ is the best site for desalination plant with *m*FAAWA approach.Again for a pessimistic perspective, the process is repeated with *m*FAAWG operator.

Step 1 Let $p=3$. Then using the *m*FAAWG operator, the values $\hat{\mathfrak {q}}_s$ for the desalination plant sites $X_s: 1\le s\le 15$ are determined as:
  $$\begin{aligned} \hat{\mathfrak {q}}_1=(0.5630,0.2098,0.2873,0.5377),&\quad \quad \hat{\mathfrak {q}}_2=(0.3653,0.2698,0.2359,0.4216),\\ \hat{\mathfrak {q}}_3=(0.4317,0.2402,0.2915,0.3848),&\quad \quad \hat{\mathfrak {q}}_4=(0.5077,0.2325,0.4172,0.3897),\\ \hat{\mathfrak {q}}_5=(0.3925,0.4113,0.1679,0.4987),&\quad \quad \hat{\mathfrak {q}}_6=(0.5208,0.2828,0.2515,0.4490),\\ \hat{\mathfrak {q}}_7=(0.2529,0.2371,0.2947,0.3192),&\quad \quad \hat{\mathfrak {q}}_8=(0.4732,0.2142,0.3764,0.4841),\\ \hat{\mathfrak {q}}_9=(0.4282,0.2505,0.2358,0.3675),&\quad \quad \hat{\mathfrak {q}}_{10}=(0.2622,0.3541,0.2349,0.1690),\\ \hat{\mathfrak {q}}_{11}=(0.2142,0.5808,0.1568,0.6003),&\quad \quad \hat{\mathfrak {q}}_{12}=(0.5135,0.2077,0.3879,0.4345),\\ \hat{\mathfrak {q}}_{13}=(0.3297,0.3717,0.3001,0.3104),&\quad \quad \hat{\mathfrak {q}}_{14}=(0.4220,0.2471,0.4771,0.4970),\\ \hat{\mathfrak {q}}_{15}=(0.3320,0.4802,0.5898,0.2473). \end{aligned}$$
Step 2 The score values S$(\hat{\mathfrak {q}}_s)$ $(\textrm{where}~ 1\le s\le 15)$ of all 4F numbers computed in previous step are calculated as:
  $$\begin{aligned} S(\hat{\mathfrak {q}}_1)&=0.3994,~\quad S(\hat{\mathfrak {q}}_2)=0.3231,~\quad S(\hat{\mathfrak {q}}_3)=0.3370,~\quad S(\hat{\mathfrak {q}}_4)=0.3868,\\ S(\hat{\mathfrak {q}}_5)&=0.3676,~\quad S(\hat{\mathfrak {q}}_6)=0.3760,~\quad S(\hat{\mathfrak {q}}_7)=0.2760,~\quad S(\hat{\mathfrak {q}}_8)=0.3870,\\ S(\hat{\mathfrak {q}}_9)&=0.3205,\quad S(\hat{\mathfrak {q}}_{10})=0.2550,\quad S(\hat{\mathfrak {q}}_{11})=0.3880,\quad S(\hat{\mathfrak {q}}_{12})=0.3859,\\ S(\hat{\mathfrak {q}}_{13})&=0.3280,\quad S(\hat{\mathfrak {q}}_{14})=0.4108,\quad S(\hat{\mathfrak {q}}_{15})=0.4123. \end{aligned}$$
Step 3 Finally, the sites are ranked as follows:
  $$\begin{aligned} X_{15}>X_{14}>X_{1}>X_{11}>X_{8}>X_4>X_{12}>X_5>X_{6}>X_3>X_{13}>X_2>X_{9}>X_{7}>X_{10}. \end{aligned}$$

Hence, $X_{15}$ is the best site for desalination plant with *m*FAAWG approach. As observed, different rankings are obtained with different AOs used. It must be noted that the tabular data utilized for both the applications is synthetic, intentionally crafted to emulate the challenges of considered site selection problems. These artificial datasets ensure a controlled evaluation environment, showcasing the algorithm’s applicability to real-world scenarios.

### Selection of a suitable site for wind power plant

Renewable energy sources have become increasingly important in recent years due to the growing need for sustainable and environment-friendly power. These green energy sources, including solar, wind, hydro, geothermal, and biomass energy, account for a clean and efficient alternative to traditional fossil fuels, which cause significant pollution and contribute to climate change. Globally, renewable energy sources now account for approximately 30% of all power generation, with China being the largest contributor to this trend. This is a significant development, as it demonstrates the growing recognition of the importance of renewable energy sources in meeting the world’s power needs.

Among these sources, wind power stands out as particularly advantageous due to its ability to generate power in an economic and environment-friendly manner. Wind turbines require minimal look-after, can work without any fossil fuels or power generators, are 100% emission-free, have no negative impact on global warming, and can be installed in various settings, making them a versatile option for power generation. Additionally, wind power is a reliable and consistent energy source even in areas with low wind speeds. Thus, wind power can help reduce pollution and combat climate change while meeting the power needs sustainably and responsibly.

Although wind power has a lot of advantages over other sources, it has some limitations, particularly regarding the appropriate siting for maximum efficiency and minimum concerns. Wind power farms need wind to operate, generate noise, and often are installable only in remote regions, increasing the transmission losses and costs. Choosing the appropriate site for a wind power plant is crucial for maximum efficiency. The efficiency of the turbines depends heavily on the wind speed and consistency in the installation area. An inappropriate location can lead to low power generation, higher maintenance costs, and reduced turbine lifespan.

Several essential factors must be considered when selecting a site for a wind power plant. Accurate measurement of wind speed and direction over an extended period is necessary to determine the site’s potential energy output. Accessibility, terrain, land use, and weather conditions should be analyzed to ensure that the site can accommodate the installation and operation of wind turbines. Another critical factor in selecting the best site is assessing potential hazards. For example, wind turbines can be threatful to birds and bats, so it is crucial to determine the local wildlife population and migration patterns. Additionally, evaluating the potential for noise pollution and visual impacts on the surrounding environment is necessary.

Suppose the government of a country is interested in the green energy plan and moving towards wind power to increase its power production while posing no harm to the environment. The geography and weather of the country support wind-power generation by its strong winds and high grounds. However, the government faces financial problems, and the project-siting must ensure minimum economic and environmental costs. For instance, keeping the project sites in remote and distant areas increases the transmission costs. Similarly, relatively adverse weather locations with stormy winds and snow can damage the turbine blades and reduce production efficiency. The site must be at a safe distance from airports and forests. In addition, placing the turbines nearer to populated areas (to minimize transmission costs) can affect the health of inhabitants (by noise and visual disturbances). Also, the site must ensure maximum efficiency with the most economic installations.

A site-selection committee, constituting meteorologists, geologists, engineers, power and finance ministry representatives, local administrations, environmental standard regulators, and the resources and approval management committee members, is assigned the task of selecting the best site while considering the challenges. The committee does an initial survey and shortlists the eight most suitable project sites ($X_i: 1\le i\le 8$). Next, the committee critically studies these shortlisted sites with the help of annual bird migration patterns, detailed weather patterns, wind directions, temperature patterns, and wind speeds. Based on these studies, the committee considers wind quality, location, power generation, environment-friendliness, and cost as the five governing parameters ($T_j: 1\le j\le 5$) for the next phase of assessments. These five parameters $T_j$ are further categorized into three sub-parameters as follows:

1. Wind quality
  (i) Good average wind speed: High annual wind speed average ensuring high power generation.
  (ii) Low speed variations: Low speed variations in different weather conditions ensure sustainability and steady output.
  (iii) High wind speed distribution: High possible wind speed distribution with the combination of wind quality and affordable infrastructure.
2. Location
  (i) Higher grounds: High plains, grounds, round tops of hills, etc., where wind is available at high speeds without interruption.
  (ii) Flat terrain: Wind turbines with giant and tall structures require smooth and flat terrains to base their structures.
  (iii) Unrestricted land availability: Site should not be a restricted zone (like near an airport) and should not have future land uses declared in the close radius.
3. Power generation
  (i) Near to the grid: Less distance from the electricity grid ensures low transmission costs and ease of installation.
  (ii) Suitability with the power flow: The newly generated power should not disturb the existing power flow and impose minimum penetration to the electrical network.
  (iii) Suitability for big turbines: Big turbines imply more power generation, but need bigger towers and hence the suitable site.
4. Environment-friendliness
  (i) Safe distance from forests: Wind turbines can be dangerous to wild life and birds, therefore a safe distance is necessary to ensure safety of these animals.
  (ii) Minimum noise pollution: Wind turbines cutting air cause noise which can affect the human health, therefore ideally site is distant from inhabited areas.
  (iii) Minimum visual impact: Site should ensure minimum shadow flickers and visual pollution.
5. Cost
  (i) Low installation cost: Different turbines work best in different conditions. Similarly near grid sites require less transmission and transformation installations. The site should ensure best working conditions with minimum installation costs.
  (ii) Affordable land cost: A site away from residential and commercial areas with suitable conditions in low cost is optimal for wind power installation.
  (iii) Low transportation cost: Connection to structured road network and accessibility minimizing the transportation costs particularly during construction, installation, and management.

Deep analysis of the sites with respect to above parameters results in a 3F decision matrix as presented in Table 2. The following weights are assigned to the parameters $T_j$ by the committee:

$$\begin{aligned} \gamma _1=0.20,\quad \gamma _2=0.15,\quad \gamma _3=0.25,\quad \gamma _4=0.10,\quad \gamma _5=0.30. \end{aligned}$$

**Table 2:** 3F decision matrix for wind power plant sites.

	$T_1$	$T_2$	$T_3$	$T_4$	$T_5$
$X_1$	(0.34, 0.76, 0.02)	(0.39, 0.71, 0.90)	(0.74, 0.68, 0.23)	(0.75, 0.81, 0.99)	(0.34, 0.97, 0.53)
$X_2$	(0.45, 0.83, 0.87)	(0.37, 0.91, 0.66)	(0.63, 0.69, 0.12)	(0.41, 0.37, 0.33)	(0.73, 0.53, 0.22)
$X_3$	(0.74, 0.19, 0.83)	(0.15, 0.09, 0.38)	(0.72, 0.62, 0.61)	(0.54, 0.13, 0.27)	(0.12, 0.34, 0.07)
$X_4$	(0.32, 0.99, 0.71)	(0.12, 0.90, 0.19)	(0.43, 0.86, 0.03)	(0.77, 0.59, 0.11)	(0.17, 0.25, 0.89)
$X_5$	(0.16, 0.56, 0.67)	(0.18, 0.42, 0.09)	(0.25, 0.91, 0.43)	(0.34, 0.66, 0.37)	(0.38, 0.11, 0.13)
$X_6$	(0.14, 0.62, 0.01)	(0.96, 0.15, 0.48)	(0.77, 0.64, 0.29)	(0.56, 0.07, 0.14)	(0.80, 0.92, 0.21)
$X_7$	(0.37, 0.12, 0.81)	(0.83, 0.21, 0.38)	(0.05, 0.13, 0.28)	(0.80, 0.52, 0.91)	(0.11, 0.42, 0.15)
$X_8$	(0.33, 0.72, 0.88)	(0.15, 0.35, 0.62)	(0.24, 0.78, 0.61)	(0.36, 0.12, 0.18)	(0.11, 0.34, 0.48)

Using Algorithm 1, the calculations to find the best site are firstly done with *m*FAAWA operator.

Step 1 Let $p=3$. Then using the *m*FAAWA operator, the values $\hat{\mathfrak {q}}_{s}$ for the wind power plant sites $X_s: 1\le s\le 8$ are calculated as:
  $$\begin{aligned} \hat{\mathfrak {q}}_1=(0.6234,0.9133,0.8969),&\quad \quad \hat{\mathfrak {q}}_2=(0.6311,0.7884,0.7101),\\ \hat{\mathfrak {q}}_3=(0.6384,0.4675,0.6680),&\quad \quad \hat{\mathfrak {q}}_4=(0.5140,0.9426,0.7872),\\ \hat{\mathfrak {q}}_5=(0.3049,0.7886,0.4982),&\quad \quad \hat{\mathfrak {q}}_6=(0.8537,0.8248,0.3183),\\ \hat{\mathfrak {q}}_7=(0.6616,0.3223,0.7348),&\quad \quad \hat{\mathfrak {q}}_8=(0.2619,0.6656,0.7347). \end{aligned}$$
Step 2 The score values S$(\hat{\mathfrak {q}}_s)$ of all 3F numbers $\hat{\mathfrak {q}}_s$ are computed as:
  $$\begin{aligned} S(\hat{\mathfrak {q}}_1)&=0.8112,\quad S(\hat{\mathfrak {q}}_2)=0.7099,\quad S(\hat{\mathfrak {q}}_3)=0.5913,\quad S(\hat{\mathfrak {q}}_4)=0.7469,\\ S(\hat{\mathfrak {q}}_5)&=0.5306,\quad S(\hat{\mathfrak {q}}_6)=0.6656,\quad S(\hat{\mathfrak {q}}_7)=0.5862,\quad S(\hat{\mathfrak {q}}_8)=0.5541. \end{aligned}$$
Step 3 Finally, the sites are ranked as follows:
  $$\begin{aligned} X_1>X_4>X_2>X_6>X_3>X_7>X_8>X_5. \end{aligned}$$

Hence, $X_1$ comes to be the best site for wind power plant.

Again, the process is repeated with *m*FAAWG aggregation.

Step 1 Let $p=3$. Then using the *m*FAAWG operator, the values $\hat{\mathfrak {q}}_s$ for the desalination plant sites $X_s: 1\le s\le 8$ are determined as:
  $$\begin{aligned} \hat{\mathfrak {q}}_1=(0.4011,0.7452,0.0961),&\quad \quad \hat{\mathfrak {q}}_2=(0.4929,0.5632,0.2168),\\ \hat{\mathfrak {q}}_3=(0.2066,0.1976,0.1619),&\quad \quad \hat{\mathfrak {q}}_4=(0.2176,0.3929,0.0979),\\ \hat{\mathfrak {q}}_5=(0.2345,0.2233,0.1829),&\quad \quad \hat{\mathfrak {q}}_6=(0.3145,0.2383,0.0608),\\ \hat{\mathfrak {q}}_7=(0.1148,0.1846,0.2484),&\quad \quad \hat{\mathfrak {q}}_8=(0.1738,0.3172,0.4169). \end{aligned}$$
Step 2 The score values S$(\hat{\mathfrak {q}}_s)$ of all 3F numbers $\hat{\mathfrak {q}}_s$ are calculated as:
  $$\begin{aligned} S(\hat{\mathfrak {q}}_1)&=0.4141,\quad S(\hat{\mathfrak {q}}_2)=0.4243,\quad S(\hat{\mathfrak {q}}_3)=0.1887,\quad S(\hat{\mathfrak {q}}_4)=0.2361,\\ S(\hat{\mathfrak {q}}_5)&=0.2136,\quad S(\hat{\mathfrak {q}}_6)=0.2045,\quad S(\hat{\mathfrak {q}}_7)=0.1826,\quad S(\hat{\mathfrak {q}}_8)=0.3026. \end{aligned}$$
Step 3 Finally, the sites are ranked as below:
  $$\begin{aligned} X_2>X_1>X_8>X_4>X_5>X_6>X_3>X_7. \end{aligned}$$

This time, $X_{2}$ comes out to be the best site for the wind power plant.

## Discussion

Aggregation operators allow to accumulate and interpret a huge data set by combining the impact of multiple related information bits into a single easily understandable entity. For decision-making with uncertain information based on a number of attributes, aggregation operators offer unique approximate solutions based on their foundational structures. This reason urges decision scientists and researchers to develop varying AOs for uncertain information systems, in order to consider multiple possible solutions effected by the trade-offs due to the varying structures of these AOs. For uncertain decision-making, these AOs are often based on T-Ns/T-CoNs customized to handle specific information. In the existing literature, multiple AOs have already been defined for *m*F information to accumulate, interpret, and appraise complicated multi-polar uncertainties. However, despite the dominating accuracy and efficient polarity demonstrated by Aczel–Alsina TN/TCoN based AOs, no work has yet established or discussed the impact of Aczel–Alsina AOs for *m*F information. Consequently, this work established *m*F Aczel–Alsina weighted AOs and demonstrated their decision-making capability with two detailed model site-selection problems. The following subsections discuss the advantages and limitations of the proposed methods shortly. In addition, comparison with some existing AOs is presented.

### Comparison

**Figure 2:**
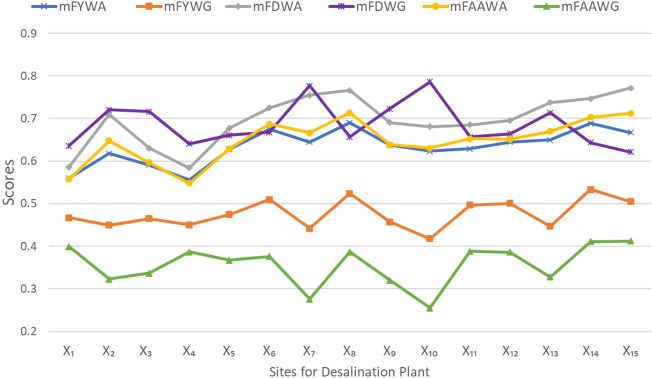
Comparison of *m*F AOs outputs in the selection of desalination plant.

Different AOs based on different TNs and TCoNs may generate different results with the same information. In order to demonstrate this variation of outcomes, previously developed *m*F weighted averaging and geometric AOs based on Yager and Dombi TNs/TCoNs have been considered. Application “Selection of suitable site for desalination plant” (Site selection for desalination plant) is taken as a test case to demonstrate this comparison. Consequently, the outcomes of proposed *m*FAAWA and *m*FAAWG AOs are compared with the outcomes of pre-existing *m*F Yager weighted averaging (*m*FYWA), *m*F Yager weighted geometric (*m*FYWG), *m*F Dombi weighted averaging (*m*FDWA), and *m*F Dombi weighted geometric (*m*FDWG) AOs. Tables 3 and 4 represent the conflicting scores and corresponding rankings with the considered AOs. This comparison is graphically represented in Fig. 2. Here, the results obtained with proposed Aczel–Alsina AOs are more inclined towards those with Yager AOs. The results with *m*FAAWA AOs are almost consistent with those obtained with *m*FYWA AOs (for instance, both declare $X_8$ as the optimal choice), however the scores with proposed averaging aggregation sandwich in between the scores with Dombi(from above) and Yager(from below) methodologies. In case of weighted geometric aggregation, *m*FAAWG AOs show significant variation (or comparative accuracy) from *m*FYWG and *m*FDWG AOs. Figure 2 shows that the aggregation scores obtained with proposed geometric aggregation demonstrate minimum variations from the weakest conjunction (every T-norm is bounded above by the minimum T-norm). The compared AOs represent significantly higher scores as compared to the minimum threshold. Another version of this observation is the considerable polarity depicted by *m*FAAWA and *m*FAAWG AOs on the basis of duality of their conjunctive and disjunctive logical bases. This polarity is least observed with Dombi AOs. Summing up, different TNs/TCoNs may generate different results with the same information. Proposed *m*FAAWA AOs show results with slight variations from the two other AOs, whereas the analyzation by *m*FAAWG AOs (TN bases AOs) is much more accurate in terms of geometric aggregation.

**Table 3:** Comparison of *m*F AOs for desalination plant site selection.

Scores$\backslash$AOs	*m*FYWA	*m*FYWG	*m*FDWA	*m*FDWG	*m*FAAWA	*m*FAAWG
$S(\hat{\mathfrak {q}}_1)$	0.5595	0.4673	0.5863	0.6360	0.5574	0.3994
$S(\hat{\mathfrak {q}}_2)$	0.6181	0.4497	0.7091	0.7205	0.6476	0.3231
$S(\hat{\mathfrak {q}}_3)$	0.5911	0.4649	0.6304	0.7162	0.5966	0.3370
$S(\hat{\mathfrak {q}}_4)$	0.5556	0.4507	0.5839	0.6406	0.5482	0.3868
$S(\hat{\mathfrak {q}}_5)$	0.6283	0.4749	0.6778	0.6610	0.6293	0.3676
$S(\hat{\mathfrak {q}}_6)$	0.6746	0.5102	0.7251	0.6676	0.6875	0.3760
$S(\hat{\mathfrak {q}}_7)$	0.6451	0.4424	0.7547	0.7774	0.6663	0.2760
$S(\hat{\mathfrak {q}}_8)$	0.6898	0.5236	0.7660	0.6570	0.7138	0.3870
$S(\hat{\mathfrak {q}}_9)$	0.6376	0.4576	0.6905	0.7235	0.6389	0.3205
$S(\hat{\mathfrak {q}}_{10})$	0.6235	0.4181	0.6812	0.7865	0.6304	0.2550
$S(\hat{\mathfrak {q}}_{11})$	0.6288	0.4971	0.6854	0.6564	0.6524	0.3880
$S(\hat{\mathfrak {q}}_{12})$	0.6453	0.5008	0.6952	0.6642	0.6510	0.3859
$S(\hat{\mathfrak {q}}_{13})$	0.6499	0.4470	0.7379	0.7132	0.6698	0.3280
$S(\hat{\mathfrak {q}}_{14})$	0.6886	0.5333	0.7465	0.6433	0.7033	0.4108
$S(\hat{\mathfrak {q}}_{15})$	0.6673	0.5054	0.7716	0.6213	0.7122	0.4123

**Table 4:** Rankings with different *m*F AOs in desalination plant site selection.

AOs	Ranking order	Choice
*m*FYWA	$X_{8}>X_{14}>X_{6}>X_{15}>X_{13}>X_{12}>X_{7}>X_9>X_{11}>X_5>X_{10}>X_2>X_{3}>X_{1}>X_{4}$	$X_8$
*m*FYWG	$X_{14}>X_{8}>X_{6}>X_{15}>X_{12}>X_{11}>X_{5}>X_1>X_{3}>X_9>X_{4}>X_2>X_{13}>X_{7}>X_{10}$	$X_{14}$
*m*FDWA	$X_{15}>X_{8}>X_{7}>X_{14}>X_{13}>X_6>X_{2}>X_{12}>X_{9}>X_{11}>X_{10}>X_5>X_{3}>X_{1}>X_{4}$	$X_{15}$
*m*FDWG	$X_{10}>X_{7}>X_{9}>X_{2}>X_{3}>X_{13}>X_{6}>X_{12}>X_{5}>X_8>X_{11}>X_{14}>X_{4}>X_{1}>X_{15}$	$X_{10}$
*m*FAAWA	$X_8>X_{15}>X_{14}>X_6>X_{13}>X_{7}>X_{11}>X_{12}>X_{2}>X_9>X_{10}>X_5>X_3>X_4>X_1$	$X_{8}$
*m*FAAWG	$X_{15}>X_{14}>X_{1}>X_{11}>X_{8}>X_4>X_{12}>X_5>X_{6}>X_3>X_{13}>X_2>X_{9}>X_{7}>X_{10}$	$X_{15}$

### Advantages

Weighted aggregation plays an important part in decision-making algorithms by integrating the effect of different parameters with their partial preference into a single entity. In addition, the *m*F sets allow accurate depiction of multi-dimensional paramterized information. This work proposed *m*F Aczel–Alsina weighted AOs and utilized these for decision-making. Some advantages and features of these AOs are listed below:

1. Proposed AOs enhance the decision-making process concerning multi-polar uncertainties by combining multi-polar efficiency of *m*F sets and accuracy of Aczel–Alsina TN.
2. Six distinct AOs offer aggregation with six different considerations. For instance, weighted averaging and weighted geometric AOs consider aggregation based on information’s existing order, whereas ordered weighted AOs make sure that preference weights are assigned based on the individual weights of the information cells.
3. Efficient polarity demonstrated by the proposed averaging and geometric AOs make them more suitable for conflicting analyses of the same information (for instance, optimistic versus pessimistic approaches).
4. The proposed AOs generate more accurate results as compared to the existing AOs. This is demonstrated in the comparative analysis.

### Limitations

Despite the advantages, proposed techniques have some limitations. One major limitation is the lengthy calculation, which increases with increasing information. This makes the procedure very difficult in case of huge information sets. In such cases, softwares like MATLAB may be used to automate the calculations. Another limitation is that different AOs may generate different results (as observed in comparison). Therefore, the results may variate from the expected outcomes.

## Conclusions and future plans

The decision-making problems are often governed by multi-polar decision parameters. *m*F sets are adequate to model such problems efficiently. Further, in order to aggregate the preferential effect of these multi-polar parameters on decision-making, many aggregation operators (AOs) have been presented in previous works. Aczel–Alsina TNs offer accurate aggregation however the literature misses any work on Aczel–Alsina AOs for *m*F information. Consequently, this work introduced novel Aczel–Alsina TN (and T-CoN) based AOs for the aggregation of *m*F information, including *m*FAAWA, *m*FAAWG, *m*FAAOWA, *m*FAAOWG, *m*FAAHWA, and *m*FAAHWG operators. Moreover, corresponding numerical examples are presented for demonstrating the developed operations and properties. For showing the MCDM capability of proposed *m*FAAWA and *m*FAAWG AOs, an algorithm is presented. Two site selection problems, including desalination plant and wind power plant are modeled and solved with the proposed algorithm for the aggregation of *m*F information. It can be observed that averaging and geometric aggregations yield different results with the same information. Finally, a comparative analysis is provided that discusses the comparison of presented *m*F Aczel–Alsina AOs with outcomes obtained by applying *m*F Yager and Dombi AOs (i.e., *m*FYWA, *m*FYWG, *m*FDWA, *m*FDWG) on the Application “Selection of suitable site for desalination plant” that considers the site selection problem of a suitable desalination plant. Similarities and variations among the compared outputs are briefly discussed in comparison section. It is observed, that despite the aggregation benefits of the proposed AOs, different results may be obtained with different AOs. Moreover, it is suggested to use powerful programs like MATLAB for implementing the algorithm on uncertain problems with huge multi-polar datasets (to ease and automate the calculations) to aid the decision-making process. The proposed work can be extended to the following directions in the coming research works:

1. *m*-polar fuzzy Aczel–Alsina prioritized aggregation operators,
2. *m*-polar fuzzy soft Aczel–Alsina aggregation operators,
3. Possibility *m*-polar fuzzy Aczel–Alsina aggregation operators,
4. Rough *m*-polar fuzzy Aczel–Alsina aggregation operators.

### Ethical approval

This article does not contain any studies with human participants or animals performed by any of the authors.

## Data Availability

The data used to support the findings of this study are included within the article.
